# A haplotype-resolved chromosome-scale genome for *Quercus rubra* L. provides insights into the genetics of adaptive traits for red oak species

**DOI:** 10.1093/g3journal/jkad209

**Published:** 2023-09-14

**Authors:** Beant Kapoor, Jerry Jenkins, Jeremy Schmutz, Tatyana Zhebentyayeva, Carsten Kuelheim, Mark Coggeshall, Chris Heim, Jesse R Lasky, Laura Leites, Nurul Islam-Faridi, Jeanne Romero-Severson, Victoria L DeLeo, Sarah M Lucas, Desanka Lazic, Oliver Gailing, John Carlson, Margaret Staton

**Affiliations:** Department of Entomology and Plant Pathology, University of Tennessee, Knoxville, TN 37996, USA; Genome Sequencing Center, HudsonAlpha Institute for Biotechnology, Huntsville, AL 35806, USA; Genome Sequencing Center, HudsonAlpha Institute for Biotechnology, Huntsville, AL 35806, USA; Department of Forestry and Natural Resources, University of Kentucky, Lexington, KY 40506, USA; Department of Ecosystem Science and Management, Pennsylvania State University, University Park, PA 16802, USA; College of Forest Resources and Environmental Science, Michigan Tech University, Houghton, MI 49931, USA; College of Agriculture, Food and Natural Resources, University of Missouri, Columbia, MO 65211, USA; Horticultural Science, North Carolina State University, Raleigh, NC 27695, USA; Department of Biology, Pennsylvania State University, University Park, PA 16802, USA; Department of Ecosystem Science and Management, Pennsylvania State University, University Park, PA 16802, USA; Forest Tree Molecular Cytogenetics Laboratory, USDA-FS, SRS-4160, Department of Ecology & Conservation Biology, Texas A&M University, College Station, TX 77843, USA; Department of Biological Sciences, University of Notre Dame, Notre Dame, IN 46556, USA; Department of Biology, Pennsylvania State University, University Park, PA 16802, USA; Department of Biology, Pennsylvania State University, University Park, PA 16802, USA; Department of Forest Genetics and Forest Tree Breeding, University of Göttingen, Göttingen, Lower Saxony 37077, Germany; Department of Forest Genetics and Forest Tree Breeding, University of Göttingen, Göttingen, Lower Saxony 37077, Germany; Department of Ecosystem Science and Management, Pennsylvania State University, University Park, PA 16802, USA; Department of Entomology and Plant Pathology, University of Tennessee, Knoxville, TN 37996, USA

**Keywords:** *Quercus rubra*, northern red oak, genome, rRNA, terpene synthase genes, plant disease resistance genes, IGT/LAZY, disease resistance genes, marcescence, bud break, quantitative trait loci, environmental adaptation, common garden, Plant Genetics and Genomics

## Abstract

Northern red oak (*Quercus rubra* L.) is an ecologically and economically important forest tree native to North America. We present a chromosome-scale genome of *Q. rubra* generated by the combination of PacBio sequences and chromatin conformation capture (Hi-C) scaffolding. This is the first reference genome from the red oak clade (section Lobatae). The *Q. rubra* assembly spans 739 Mb with 95.27% of the genome in 12 chromosomes and 33,333 protein-coding genes. Comparisons to the genomes of *Quercus lobata* and *Quercus mongolica* revealed high collinearity, with intrachromosomal structural variants present. Orthologous gene family analysis with other tree species revealed that gene families associated with defense response were expanding and contracting simultaneously across the *Q. rubra* genome. *Quercus rubra* had the most CC-NBS-LRR and TIR-NBS-LRR resistance genes out of the 9 species analyzed. Terpene synthase gene family comparisons further reveal tandem gene duplications in TPS-b subfamily, similar to *Quercus robur*. Phylogenetic analysis also identified 4 subfamilies of the IGT/LAZY gene family in *Q. rubra* important for plant structure. Single major QTL regions were identified for vegetative bud break and marcescence, which contain candidate genes for further research, including a putative ortholog of the circadian clock constituent cryptochrome (CRY2) and 8 tandemly duplicated genes for serine protease inhibitors, respectively. Genome–environment associations across natural populations identified candidate abiotic stress tolerance genes and predicted performance in a common garden. This high-quality red oak genome represents an essential resource to the oak genomic community, which will expedite comparative genomics and biological studies in *Quercus* species.

## Introduction

Northern red oak (*Quercus rubra* L.), an economically and ecologically important tree species in North America, is a member of the *Quercus* genus in the Fagaceae family. It is a valuable source of hardwood lumber, often used for veneer, flooring, furniture, and high-quality firewood (Millers et al. 1989; Sork et al. 1993). *Quercus rubra* can withstand dry, acidic soil conditions as well as air pollution and can also be salt tolerant (Bisgrove 2010; Nesom 2001). *Quercus rubra* is the dominant, keystone tree species in many forest types across its native range, and its acorns are consumed by many native wildlife species, particularly after replacing the ecological role of the American chestnut (*Castanea dentata*) (Vengadesan and Pijut 2009). It has a wide geographic range, from the Nova Scotia peninsula to Minnesota and south to Alabama and North Carolina (Sander 1990). Ecosystem services include soil protection/stabilization and improvement, carbon sequestration, and acting as a windbreak (plain areas, continental sand dunes) and as a fire belt (especially in pine regions) (Dold et al. 2019; Ontl et al. 2020; Satvi et al. 2022). While *Q. rubra* can grow in various soil and topographic conditions, often establishing monotypic stands (Little 1979; Sander 1990), they grow best on lower and middle slopes with well-drained valley floors (Sander 1990). They have developed mechanisms of drought tolerance, drought avoidance (such as deeper tap roots and triggered stomatal closure), or both (Rauschendorfer et al. 2022). With characteristics such as high levels of heterozygosity, cohabitation, an outcrossing mating system, and a wide geographical range, *Q. rubra* is an excellent model tree species for the study of gene flow, local adaptation, speciation, and population genomics (Alexander and Woeste 2014; Leites et al. 2019; Lind-Riehl and Gailing 2015; Makela et al. 2016; Oyama et al. 2017; Rodríguez-Correa et al. 2018; Soltani et al. 2020).

Precipitously declining forest health has serious short- and long-term ecological and economic implications for many forest tree species, including oaks. Genomic resources and molecular tools for tree improvement and management programs are urgently required (Neale and Kremer 2011). Chromosome-scale assemblies for species such as *Quercus robur* (Plomion et al. 2018), *Quercus lobata* (Sork et al. 2022), and *Quercus mongolica* (Ai et al. 2022) are publicly available, and numerous transcriptome studies have also been conducted in oak species (Gugger et al. 2017; Le Provost et al. 2016; Magalhães et al. 2016; Soltani et al. 2020). Some white oak genome studies report a high number of resistance (NBS-LRR) genes, hypothesized to be associated with their longevity and the perpetual coevolutionary “arms race” with pests and pathogens (Plomion et al. 2018; Sork et al. 2022). Other gene families of interest include terpene synthases, which serve key functions in the growth and development of plants as well as in regulating plant interactions with the environment (Jiang et al. 2019; Kulheim et al. 2015), and IGTs, which are the main controllers of the architecture in trees (Waite and Dardick 2021). The IGT family controls the gravitropic set-point angle (GSA), i.e. the angle of maintenance of lateral shoots and roots relative to gravity. Branch angle is a key shoot architecture trait that may influence photosynthesis efficiency, tree canopy size, tree height, and wood quality (Ehleringer and Werk 1986; Ezcurra et al. 1991; Glenn et al. 2015; Hill and Hollender 2019). IGTs have been found to play a role in tree architecture of peach, apple, poplar, and orange trees (Dougherty et al. 2023; Dutt et al. 2022; Fladung 2021; Waite and Dardick 2021).

In addition to biotic stressors, abiotic stress is increasingly impacting forest health. Ascertaining the gene networks and heritable loci underlying these traits could quickly advance tree genetic improvement efforts (Dudley and Moll 1969; Fasoula and Fasoula 2002; Murray et al. 2008). Several studies suggest that changing climatic conditions such as warmer spring temperatures have advanced the timing of bud break in many plant species (Ellwood et al. 2013; Schwartz et al. 2006), and that bud break is strongly genetically controlled (Abbott et al. 2015; Falavigna et al. 2019; Singh et al. 2018). Oaks also display marcescence (Mc), a phenomenon in which leaves senesce in the fall but the abscission layer does not form. Senescent leaves are retained on the branches into the spring season. Mc is theorized to be an adaptive trait that produces a new annual layer of mulch surrounding the tree, providing nutrients for growth during and protecting the root system from drought (ABADíA et al. 1996; Vila-Viçosa et al. 2020). Alternative theories include an improvement in nutrient reabsorption during senescence or protection of overwintering buds from water loss or frost (Heberling and Muzika 2023). These and other theories remain largely untested, despite potential implications for the role of Mc in response to climate change.

While a dense genetic map is available for *Q. rubra* (Konar et al. 2017), no reference genome yet exists. Despite the sympatry of many oak species in the white oak and red oak clades, they differ in reproductive strategies (1- vs 2-year acorn maturation), wood structure (constitutive tylose induction), disease resistance (oak wilt susceptibility), leaf morphology (bristle tips), and more. Genomic resources across sections of the *Quercus* genus are needed to explore the evolutionary basis of these traits. This study aims to close this gap by producing a haplotype-resolved, chromosome-scale assembly to provide genetic and genomic resources for *Q. rubra*.

## Materials and methods

Additional methodology details are available in the Supplementary Methods.

### Reference genome assembly and annotation

A *Q. rubra* tree from a 3-generation pedigree (West Lafayette, IN, USA) was selected for the reference genome assembly and annotation (Supplementary Methods 1.1). High molecular weight DNA was extracted from 10 g each of dormant leaf bud and twig bark tissues for Illumina, PacBio, and Hi-C sequencing (Supplementary Methods 1.2 and 1.3). The haplotype-resolved *Q. rubra* genome assembly and annotation were generated by HudsonAlpha Biotechnology Institute (HAI) (Huntsville, AL, USA) (Supplementary Methods 1.4 and 1.5) and is available in Phytozome (Goodstein et al. 2012). Chromosomes were oriented and numbered using the publicly available *Q. rubra* linkage map (Konar et al. 2017). To validate the correspondence of the 12 longest *Q. rubra* assembly chromosomes, we aligned an existing linkage map consisting of 957 sequence markers (mostly SNPs) to our primary *Q. rubra* assembly (genome hereafter) (Konar et al. 2017) (Supplementary Methods 1.4). The completeness of *Q. rubra* genome and alternate haplotype assembly were assessed using the Benchmarking Universal Single-Copy Orthologs (BUSCO) v5.0 against the *embryophyta_odb10* data set (Seppey et al. 2019). The long terminal repeat (LTR) assembly index (LAI), a standard for evaluating repeat sequence assembly, was also used to assess assembly contiguity (Ou et al. 2018). The protein sequences of *Q. rubra* genome were aligned to that of alternate haplotype with BLASTP 2.11.0+ (Altschul et al. 1990). The unique best hits from the blast results were visualized using Circos v0.69-8 (Krzywinski et al. 2009). We used Sniffles v2.0.7 to call structural variants (SVs) (Sedlazeck et al. 2018) (Supplementary Methods 1.4). The location of 5S, 18S, and 28S rDNA subunits in the *Q. rubra* genome was characterized using fluorescence in situ hybridization (FISH) with rDNA oligonucleotide probes in vivo and RNAmmer v1.2 (Lagesen et al. 2007) (Supplementary Methods 1.5 and 1.6).

### Comparative genomic analyses

We used OrthoFinder v2.5.2 (Emms and Kelly 2019) to identify gene orthogroups between *Q. rubra* and 7 other plant species: *C. dentata* v1.1, *Castanea mollissima* (Wang et al. 2022), *Cucumis sativus* v1.0, *Prunus persica* v2.1 (Verde et al. 2013), *Q. robur* (Plomion et al. 2018), *Q. lobata* (Sork et al. 2022), and *Q. mongolica* (Ai et al. 2022) (Supplementary Methods 1.7). The orthologous gene families and phylogenetic tree topology inferred from OrthoFinder were input to CAFE5 to identify significant expansion or contraction in each gene family (*P* < 0.01) (Mendes et al. 2020) (Supplementary Methods 1.7). Enrichment analysis on these sets of genes was performed in Biology Network Gene Ontology tool (BINGO) v3.0.3 (Maere et al. 2005) using *Q. rubra* custom annotation file as reference provided by EnTAP v0.10.8 (Hart et al. 2020). We used Mummer4 and Syri v1.6 to profile structural variation (SV) between *Q. rubra* genome, *Q. lobata*, and *Q. mongolica* with high-quality, chromosome-scale genomes (Ai et al. 2022; Goel et al. 2019; Marçais et al. 2018; Sork et al. 2022) (Supplementary Methods 1.7).

### Terpene synthase, IGT, and disease resistance gene family comparisons

Terpene synthase gene families (TPS; PFAM: PF03936 and PF01397) and IGT plant architecture genes from *Q. rubra* and 8 other species (*Arabidopsis thaliana*, *Eucalyptus grandis*, *Populus trichocarpa*, *Vitis vinifera*, *Q. robur*, *Carica papaya*, *P. persica*, and *Theobroma cacao*) were identified and compared (Kulheim et al. 2015; Waite and Dardick 2021) (Supplementary Methods 1.8). Using the Disease Resistance Analysis and Gene Orthology (DRAGO2) pipeline (Osuna-Cruz et al. 2018), protein sequences from the same 8 species were used to identify plant disease resistance–related domains and gene families (R-genes) (Supplementary Methods 1.8).

### Leaf emergence and Mc QTL regions

The F_1_ progeny (SM1 × SM2) that served as the basis for the dense genetic map (Konar et al. 2017) was scored for leaf emergence (LE; bud break), as reported by Heim (2016). Using protocols previously reported for *Q. robur* (Scotti-Saintagne et al. 2004), a total of 205 ramets of 89 full-sibs in the *Q. rubra* mapping population were phenotyped for the date of spring leafing over 3 consecutive years (2014–2016). In April 2021, 71 full-sibs, representing 48 clones, were again phenotyped for date of spring leafing, using the same protocol as reported by Heim (2016). Additionally, a total of 5 grafts representing both parents (SM1 and SM2) were also scored for bud break in the same planting. The same full-sib population was phenotyped for Mc as previously described (Heim 2016). Briefly, Mc phenotyping was performed in February of 2015 and 2016 using a 0–5 scale of leaf retention. A rating of 0 indicated no leaves present, while a rating of 5 indicated all leaves were retained on the tree, i.e. fully marcescent. QTL mapping for both traits was conducted using the approximate multiple QTL model (MQM) implemented in MapQTL (van Ooijen et al. 2000). The minimum LOD score for QTL detection was determined by the genome-wide LOD significance threshold (*α* = 0.05) calculated using 1,000 permutations. QTL (q) names reflected the trait (i.e. LE and Mc) appended with the chromosome associated with the trait. Candidate genes were identified within the LE QTL region and compared to QTL regions associated with the chill requirement and bloom date in *P. persica* (Supplementary Methods 1.9).

### Population structure and local adaptation

In July 2016, we collected leaf tissues from 78 individuals at a *Q. rubra* provenance trial in Vallonia, IN, USA, planted in 1991 (Coggeshall 1993). The 78 individuals included 3 seedlings (i.e. maternal half-siblings) from each of the 26 original seed parent trees, each “seedling” (now grown trees) selected from replicates at different locations in the common garden. We also collected leaf tissues from 18 seedlings (2 individuals each per 9 provenances) obtained from Pennsylvania (PA) *Q. rubra* progeny trials in the PA Bureau of Forestry nursery, for a total of 96 individuals from wild-collected seed. Overall, the selected provenances covered a north–south transect of over 1,400 km, including 4 populations sampled from the northeastern extreme of the range (in southern Ontario and Quebec at ∼45.5°N and 76.5°S), 5 populations sampled from the southeastern part of the range (in the Appalachian Mountain zone of Georgia, North Carolina, and Tennessee at ∼35.5°N and 83.5°S), and 4 populations across northern PA and 5 populations across southern PA representing a transition region from southern to northern provenances at ∼41°N. Exome capture data were produced and processed to identify high-quality biallelic SNPs, leaving 93 accessions after filtering (Supplementary Methods 1.10).

Pairwise genetic distances between samples and population structure in the data set were accessed using PLINK followed by multidimensional scaling of these distances in 2 dimensions (Purcell et al. 2007). To identify individual loci associated with local adaptation, we used 2 complementary approaches. First, we used the R package “hierfstat” (Goudet 2005) to calculate *F*_ST_ values between the 4 sampled geographic population clusters: Southern Appalachian, Indiana, PA, and Canada. Second, we implemented redundancy analysis (RDA), a multivariate ordination approach that maximizes the proportion of SNP variation explained by linear combinations of environmental variables (Forester et al. 2018). We phenotyped trees in July 2016 at the sampled common garden in Vallonia, IN. We measured height and diameter at breast height (DBH) to assess performance and scanned leaves with a LI-3100C leaf area meter (LI-COR) (Supplementary Methods 1.10).

### Estimation of nucleotide diversity

Mature leaves from 60 *Q. rubra* trees were sampled in spring 2020 at 2 different locations in the United States. Thirty samples were collected at Baraga Plains, MI, USA (46.64°N and −88.52°W), and 30 samples were collected at Lisle, IL, USA (41.81°N and −88.05°W). DNA was extracted from fresh leaves using a modified CTAB extraction protocol (Kubisiak et al. 2013). Sequencing was performed on these DNA samples at BGI using the DNBSEQ platform with an expected target coverage of 30×. After SNP calling and filtering (Supplementary Methods 1.11), the final data set for nucleotide diversity calculation consisted of 51.8 million positions including monomorphic sites. For *F*_ST_ outlier detection analysis, only biallelic sites were kept, and a minor allele frequency (MAF) filter of 0.05 was applied to generate the data set of 5.9 million SNPs (Supplementary Methods 1.11).

## Results

### Genome assembly and annotation

We generated a chromosome-level genome assembly of *Q. rubra* with an assembly of length 739.58 Mb, a scaffold N50 value of 58.1 Mb, and a contig N50 value of 1.92 Mb (Table 1). This genome assembly is comparable in contiguity to recently published oak species, such as *Q. lobata* (version 3.0) (scaffold N50 = 66.42 Mb; contig N50 = 247 Kb) (Sork et al. 2022) and *Q. mongolica* (scaffold N50 = 66.74 Mb; contig N50 = 2.64 Mb) (Ai et al. 2022). The 12 chromosomes account for 95.27% of the total *Q. rubra* genome length and were named and oriented against the genetic map (Konar et al. 2017). The GC content of the *Q. rubra* genome, 34.83% (Table 1), was very similar to that of *Q. robur* (35.65%) and *Q. lobata* (35.41%).

**Table 1.** Summary of the primary and alternate haplotype assembly of *Q. rubra* genome.

	Primary assembly (all)	Primary assembly (chromosomes)	Alternate haplotype
Number of scaffolds	966	12	12
Number of contigs	1,593	639	3,662
Total length (bp)	739,579,365	704,594,486 (95.27%)	623,822,028
Largest scaffold (bp)	90,623,836	90,623,836	74,645,423
Smallest scaffold (bp)	5,010	39,442,637	34,313,110
Number of Ns	6,270,000 (0.85%)	6,270,000 (0.85%)	36,500,000 (5.85%)
Scaffold N50 (bp)	58,110,601	59,510,927	56,112,388
Scaffold L50	6	5	5
Contig N50 (bp)	1,924,460	2,021,542	227,488
Contig L50	109	100	774
Scaffold N90 (bp)	39,442,637	43,114,498	40,063,876
Scaffold L90	12	11	11
Contig N90 (bp)	345,247	530,059	74,352
Contig L90	434	361	2522
GC (%)	34.83%	34.81%	34.73%
Repetitive elements (%)	50.15%	47.51%	46.26%
Protein-coding gene models	33,333	31,784 (95.35%)	29,265
Predicted protein sequences	47,780	45,928 (96.12%)	40,389
Complete BUSCOs (C)	1,577 (97.71%)	1,565 (97%)	1,303 (80.73%)
Complete and single-copy BUSCOs (S)	1,493 (92.5%)	1,499 (92.9%)	1,242 (76.95%)
Complete and duplicated BUSCOs (D)	84 (5.2%)	66 (4.1%)	61 (3.78%)
Fragmented BUSCOs (F)	18 (1.12%)	17 (1.1%)	60 (3.7%)
Missing BUSCOs (M)	19 (1.18%)	32 (1.9%)	251 (15.5%)

**Fig. 1.**
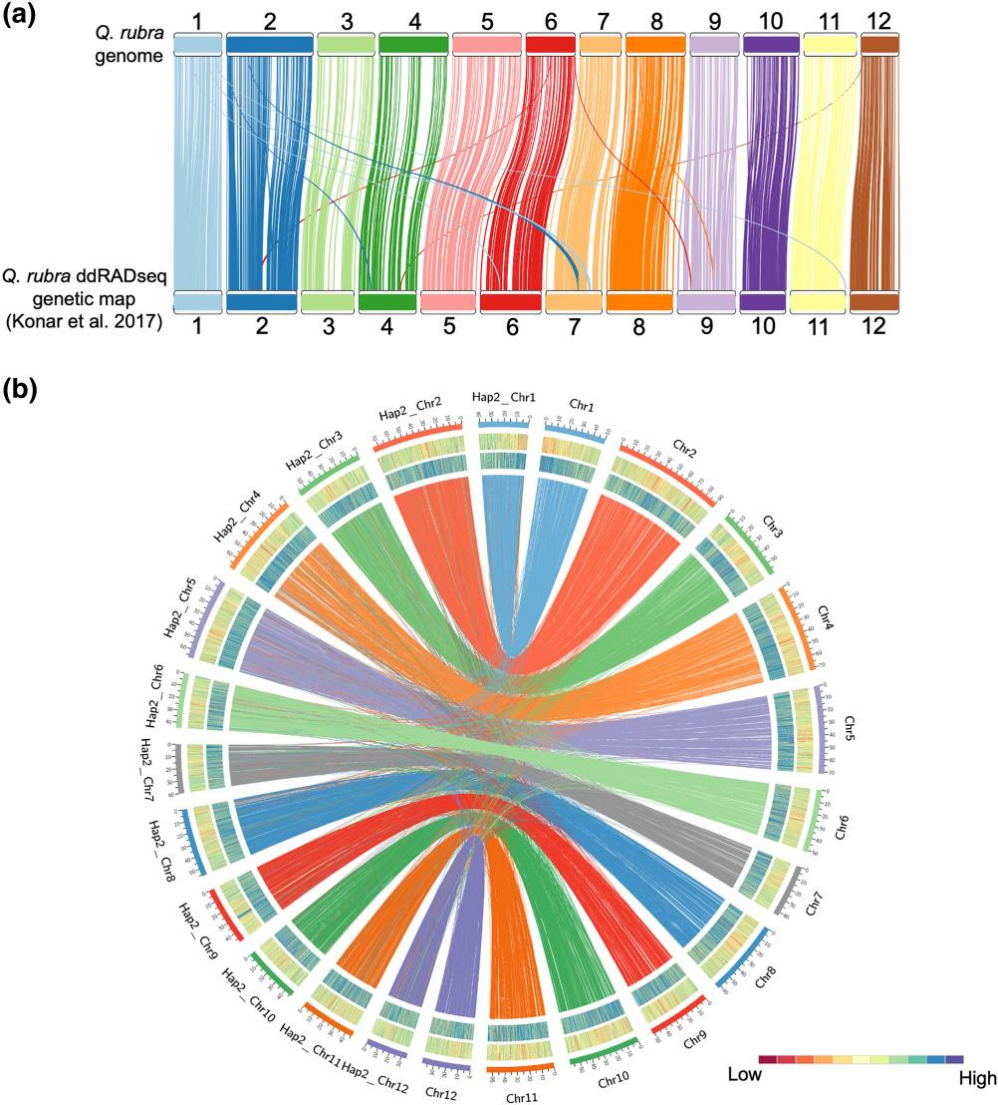
a) Alignment of the sequence-based genetic markers from the high-resolution genetic map (bottom) to the chromosomes of the *Q. rubra* genome (top). b) Outer track—repetitive element density (percentage). Inner track—gene density (percentage). Lines between chromosomes of primary assembly and alternate haplotype indicate synteny links. The primary assembly is named Chr1 to Chr12; the alternate haplotype is named Hap2_Chr1 to Hap2_Chr12.

To evaluate the quality of the *Q. rubra* genome, the sequences for each marker from a high-resolution linkage map (Konar et al. 2017) were aligned to the genome to assess the accuracy of the original contigs and scaffolding into chromosome order. Out of 952 markers with sequences available, 849 (89.18%) mapped uniquely to the *Q. rubra* genome, 46 (4.83%) to 2 locations, 32 (3.36%) to more than 2 locations, and 25 (2.63%) were unmapped (Fig. 1a), and 16 (1.68%) mapped to unplaced scaffolds in the *Q. rubra* genome. We found a predominantly monotonic 1-to-1 correspondence between linkage groups of the genetic map and the 12 largest scaffolds of our assembly and thus renamed our scaffolds as chromosomes, adopting the linkage group (LG) numbering and orientation of the genetic linkage map. The assembly was further evaluated by mapping paired-end short reads to determine the proportion of the genome captured in the assembly. Over 91% of short-read pairs map concordantly to the genome. BUSCO analysis indicated that 97.71% of the core embryophyte genes were completely present in the *Q. rubra* genome, out of which 92.5% genes were single copy and 5.2% were duplicated (Table 1) (Seppey et al. 2019). We found 84 duplicated BUSCO genes in *Q. rubra* genome whereas there were 145 duplicated BUSCO genes present in the *Q. lobata* and *Q. mongolica* genomes (Ai et al. 2022; Sork et al. 2022). Twenty-six BUSCOs were duplicated in all 3, suggesting that these genomic duplications originated during the evolution of *Quercus* lineage and are not the result of the assembly artifacts. The completeness of nongenic regions of the assembly was assessed by examining LTR retrotransposon structure. The *Q. rubra* genome assembly had an LAI score of 17.49, which is typical of a reference-quality genome (Ou et al. 2018). Overall, all evaluation metrics indicated that the assembly was largely complete and properly scaffolded.

The genome was not fully phased during assembly. To determine haploblocks, phasing with long-read sequences using WhatsHap v1.4 was conducted (Martin et al. 2016). Of the 4,967,526 heterozygous single nucleotide variants (SNVs), 4,951,851 (99.7%) were phased into 1,730 haploblocks. The blocks varied in length from 10 bp to 5.99 Mb with an average block size of 398.52 kb (Supplementary Table 1 in Supplementary File 2).

The 50.15% of the genome identified as repetitive included a high number of LTRs, primarily from the Ty1-Copia (9.13%) and Ty3-Gypsy (15.54%) families (Table 1). Gene annotation yielded 33,333 protein-coding genes and 47,780 protein-coding transcripts. 95.35% of protein-coding genes were located on the 12 chromosomes. After functional annotation, 12,213 transcripts had at least 1 pathway assignment from KEGG, and 29,721 transcripts were assigned to at least 1 Gene Ontology (GO) term. 83.29% of transcripts were annotated with a sequence similarity match to a protein database, and 94.82% were associated with at least 1 Protein Analysis Through Evolutionary Relationships (PANTHER) term.

### Alternate haplotype assembly and annotation

An alternate haplotype was assembled with a scaffold N50 value of 56.11 Mb and a contig N50 value of 227.49 kb. The length of the alternate haplotype is significantly shorter than the reference assembly at 623.82 Mb with 80.73% complete BUSCO genes, out of which 76.95% were single-copy genes and 3.78% were duplicated (Table 1). Annotation masked 46.26% of the assembly as repetitive and identified 29,265 protein-coding genes. Alignment of the protein sequences of *Q. rubra* genome assembly and the alternate haplotype revealed overall high collinearity with small gaps and inversions (Fig. 1b). To better understand the SV present in the genome of this diploid individual, the PacBio long reads were mapped to the genome assembly. We found 1,946 well-supported SVs > 10 kb and <1 Mb. These SVs included 75 inversions, 528 insertions, 1,197 deletions, and 146 duplications.

### rRNA characterization

**Fig. 2.**
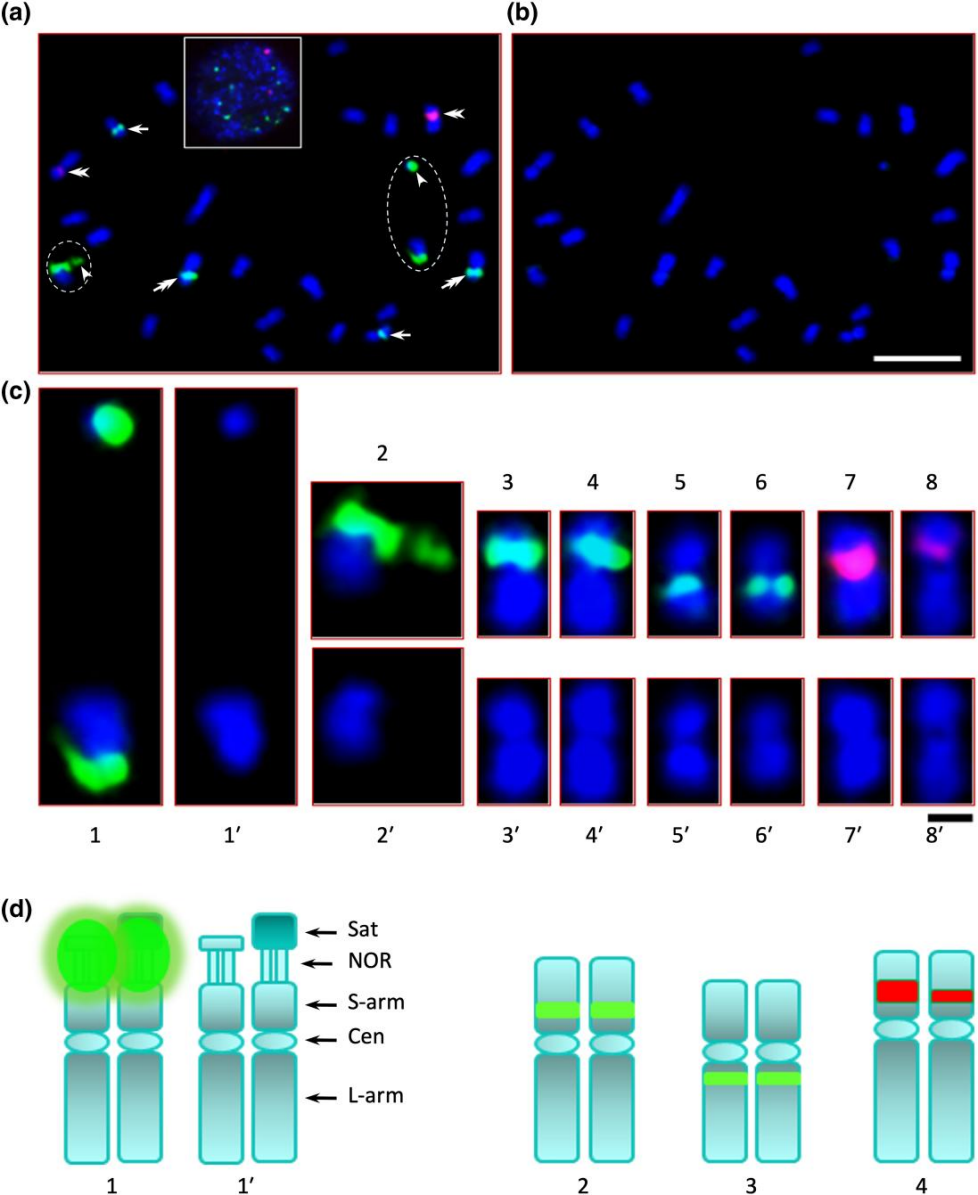
Somatic chromosome spread of *Q. rubra* seedling root tips FISHed with 18S–5.8S and 5S rDNA oligosynthesized probes. a) Superimposed image of red–green–blue filters, showing 3 pairs of chromosomes with 35S sites (green signals) and 1 pair with 5S site (red signal), and an interphase nucleus is shown in the insert. b) DAPI image of the same chromosomes as in a). c) Individual pairs of rDNA-bearing chromosomes. d) Diagrammatic representation of the 4 rDNA-bearing chromosomes. In c), c1 and c2 are homologous pairs with the major 35S rDNA signal showing the detached NOR, and c1′ and c2′ are the DAPI images, respectively; c3 and c4 are homologous pairs with the medium 35S rDNA signal, and c3′ and c4′ are the DAPI images, respectively; c5 and c6 are homologous pairs with the minor 35S rDNA signal, and c5′ and c6′ are the DAPI images, respectively; and c7 and c8 are homologous pairs with the 5S rDNA signal, and c7′ and c8′ are the DAPI images, respectively. In d), d1 and d1′ are diagrammatic representations of the major 35S bearing chromosome pair with and without the 35S (green) signal, respectively; d2 is the representation of the medium 35S containing chromosome pair; d3 is the representation of the minor 35S containing chromosome pair; and d4 is the representation of the 5S containing chromosome pair. Note: The 5S homologous pair is possibly the second or third largest pair in the complement, the 35S-md (35S medium) pair is possibly the third or fourth largest pair, and the 35S-mn (35S minor) pair is one of the smallest pairs. The scale bar in b) is 5 µm and in c) is 1 µm.

Four regions were identified with 18S–5.8S–26S rRNA genes (35S array) in the *Q. rubra* genome: chr 1 at 200 kb, chr 1 at 2.9 Mb, chr 4 at 47.8 Mb, and chr 11 at 29 Mb (Supplementary Table 2 in Supplementary File 2). The 5S subunit was found at a single location, chr 5 at 45 Mb. We explored these results further with FISH using 18S/5.8S and 5S synthetic oligonucleotide probes on *Q. rubra* chromosome spreads. We observed 3 35S and 1 5S rDNA sites, located independently on 4 different pairs of chromosomes (Fig. 2). The 3 35S were identified as a major locus located terminally on a homologous pair and a medium and minor locus located proximally in pericentromeric regions. The 5S locus was located in a pericentromeric region.

### Comparative genomic analysis

Orthogroups were predicted from *Q. rubra*, 5 additional species from the Fagales (*Q. lobata*, *Q. mongolica*, *Q. robur*, *C. mollissima*, and *C. dentata*), and 2 outgroups (*C. sativus* and *P. persica*). This yielded a total of 26,679 orthologous gene groups (orthogroups) that comprised 244,271 genes (Table 2). Among these gene families, 8,830 orthogroups were shared by all 8 species. A total of 3,192 orthogroups containing 12,765 genes were species specific (Table 2). For *Q. rubra*, 32,352 (97.06%) genes were assigned to 19,715 orthogroups; 183 orthogroups and 529 genes were species specific. Based on orthogroup membership, *Q. rubra* has 188 expanded and 186 contracted gene families (*P* < 0.01) (Supplementary Fig. 1 in Supplementary File 1 and Supplementary Tables 3 and 4 in Supplementary File 2). To explore the function of these gene families, GO term enrichment was performed and identified 428 significantly enriched GO terms for expanding gene families and 667 for contracting families. The top 5 enriched GO terms in expanding gene families were defense response (GO ID: 6952, p_adj = 1.07E-61), multiorganism process (GO ID: 51704, p_adj = 1.53E-44), response to stress (GO ID: 6950, p_adj = 2.70E-40), transferase activity (GO ID: 16772, p_adj = 1.37E-36), and nucleotide binding (GO ID: 166, p_adj = 2.22E-36) (Supplementary Table 3 in Supplementary File 2). The top 5 enriched GO terms in contracting gene families were adenyl ribonucleotide binding (GO ID: 32559, p_adj = 9.04E-39), diacylglycerol binding (GO ID: 19992, p_adj = 2.44E-38), adenyl nucleotide binding (GO ID: 30554, p_adj = 3.11E-38), purine ribonucleotide binding (GO ID: 32555, p_adj = 1.24E-35), and ribonucleotide binding (GO ID: 32553, p_adj = 5.38E-35). Interestingly, the defense response GO term (GO ID: 6952) was enriched in both expanding and contracting gene families in the *Q. rubra* genome (Supplementary Table 4 in Supplementary File 2).

**Table 2.** Statistics of genes assigned to orthogroups between *Q. rubra* and 7 tree species from the rosid I clade (*Q. robur*, *Q. lobata*, *C. dentata*, *C. mollissima*, *C. sativus*, and *P. persica*).

Species	*Q. rubra*	*Q. robur*	*Q. lobata*	*Q. mongolica*	*C. dentata*	*C. mollissima*	*C. sativus*	*P. persica*
Total number of genes	33,333	25,808	39,373	36,553	31,254	33,597	30,919	26,873
Number of orthogroups	19,715	15,370	21,342	20,132	19,611	19,275	14,549	16,356
Number of genes in orthogroups	32,352	24,252	37,411	34,261	29,891	32,597	29,378	24,129
Number of unassigned genes	981	1,556	1,962	2,292	1,363	1,000	1,541	2,744
Percentage of genes in orthogroups	97.1	94	95	93.7	95.6	97	95	89.8
Percentage of unassigned genes	2.9	6	5	6.3	4.4	3	5	10.2
Number of species-specific orthogroups	183	200	500	563	138	267	766	575
Number of genes in species-specific orthogroups	529	534	1,369	3,117	376	1,144	3,172	2,524
Percentage of genes in species-specific orthogroups	1.6	2.1	3.5	8.5	1.2	3.4	10.3	9.4

**Fig. 3.**
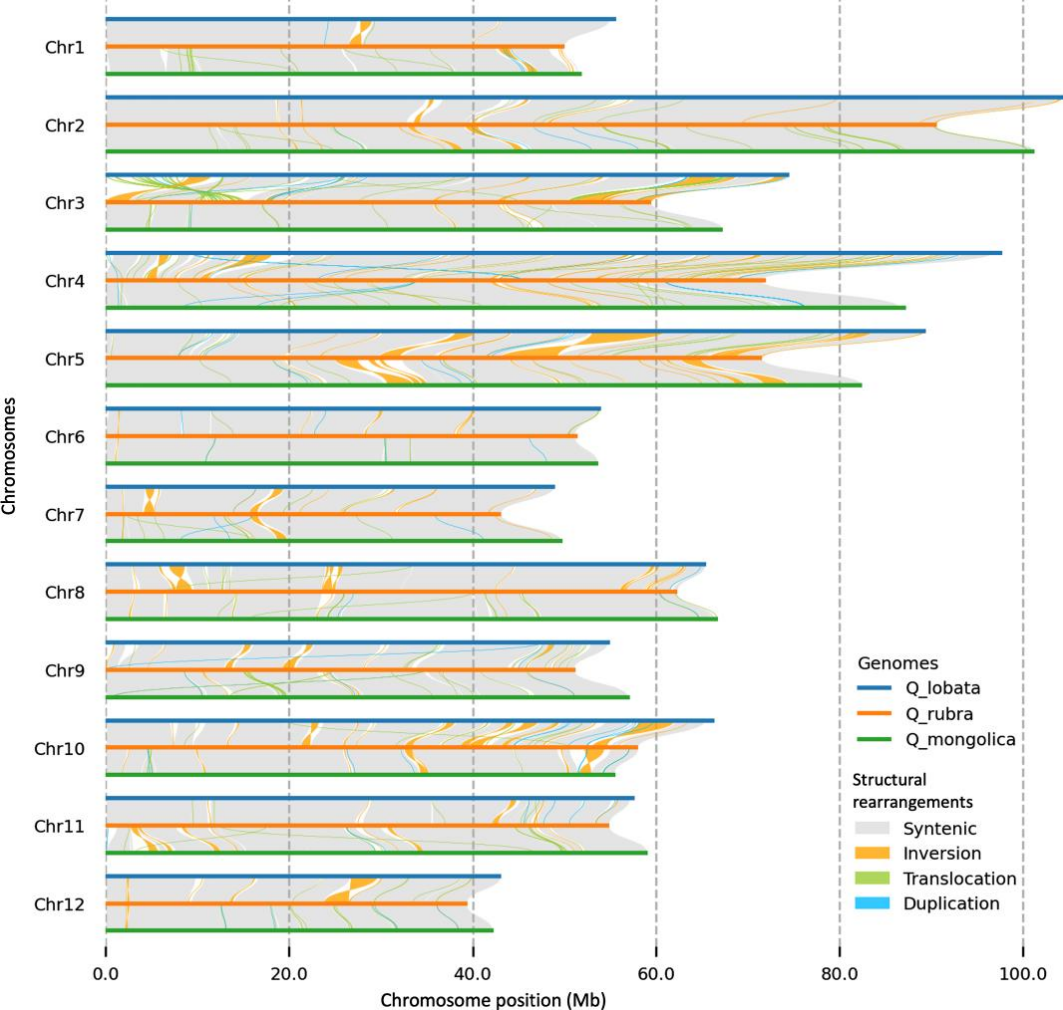
Identification of synteny (gray) and structural rearrangements (inversions, translocations, and duplications) between the chromosomes of *Q. lobata* (top), *Q. ru*b*ra* (middle), and *Q. mongolica* (bottom). Chromosomes are numbered the same across all species, but chromosomes 1, 5, 6, 10, and 11 were reverse complemented in *Q. lobata* and *Q. mongolica* to be in the same orientation as *Q. rubra*, which adheres to its linkage map orientation.

We compared our *Q. rubra* genome to the available high-quality, chromosome-level white oak clade genomes, *Q. lobata* and *Q. mongolica*, using Syri v1.6 and Mummer4. Mummer4 revealed high similarity between the 3 species, despite ∼47 million years since divergence from a common ancestor (Grímsson et al. 2016). *Quercus lobata* and *Q. mongolica* share 95.36% identity whereas the identity of the *Q. rubra* genome to the *Q. lobata* and *Q. mongolica* genomes was 91.65 and 91.78%, respectively (Supplementary Fig. 2 in Supplementary File 1). Despite an overall high level of synteny across the 12 chromosomes, Syri found structural rearrangements when *Q. rubra* was compared to both the *Q. lobata* and *Q. mongolica* genomes. We identified 233 inversions between *Q. rubra* and *Q. lobata* ranging from 295 bp in chr 8 to 6.23 Mb in chr 5 (Fig. 3; Supplementary Table 5 in Supplementary File 2). There were 163 inversions present between the *Q. rubra* and *Q. mongolica* genomes ranging from 288 bp in chr 2 to 2.42 Mb in chr 5 (Fig. 3; Supplementary Table 5 in Supplementary File 2). There were 13 inversions greater than 1 Mb between *Q. rubra* and *Q. lobata*, whereas there were only 5 inversions greater than 1 Mb between *Q. rubra* and *Q. mongolica*. As would be expected from the phylogeny with the 2 white oaks having a more recent common ancestor, some of the inversions were shared for both *Q. lobata* and *Q. mongolica*. For example, an inversion of 900 kb on chr 7 was present between the *Q. rubra*, *Q. lobata*, and *Q. mongolica* genomes at around 15.69 Mb (Fig. 3). Furthermore, a 750 kb inversion was observed on chr 10 in *Q. rubra* when compared to both *Q. lobata* and *Q. mongolica* at 32.59 Mb (Fig. 3).

### Terpene synthase, IGT, and disease resistance gene family comparison

**Fig. 4.**
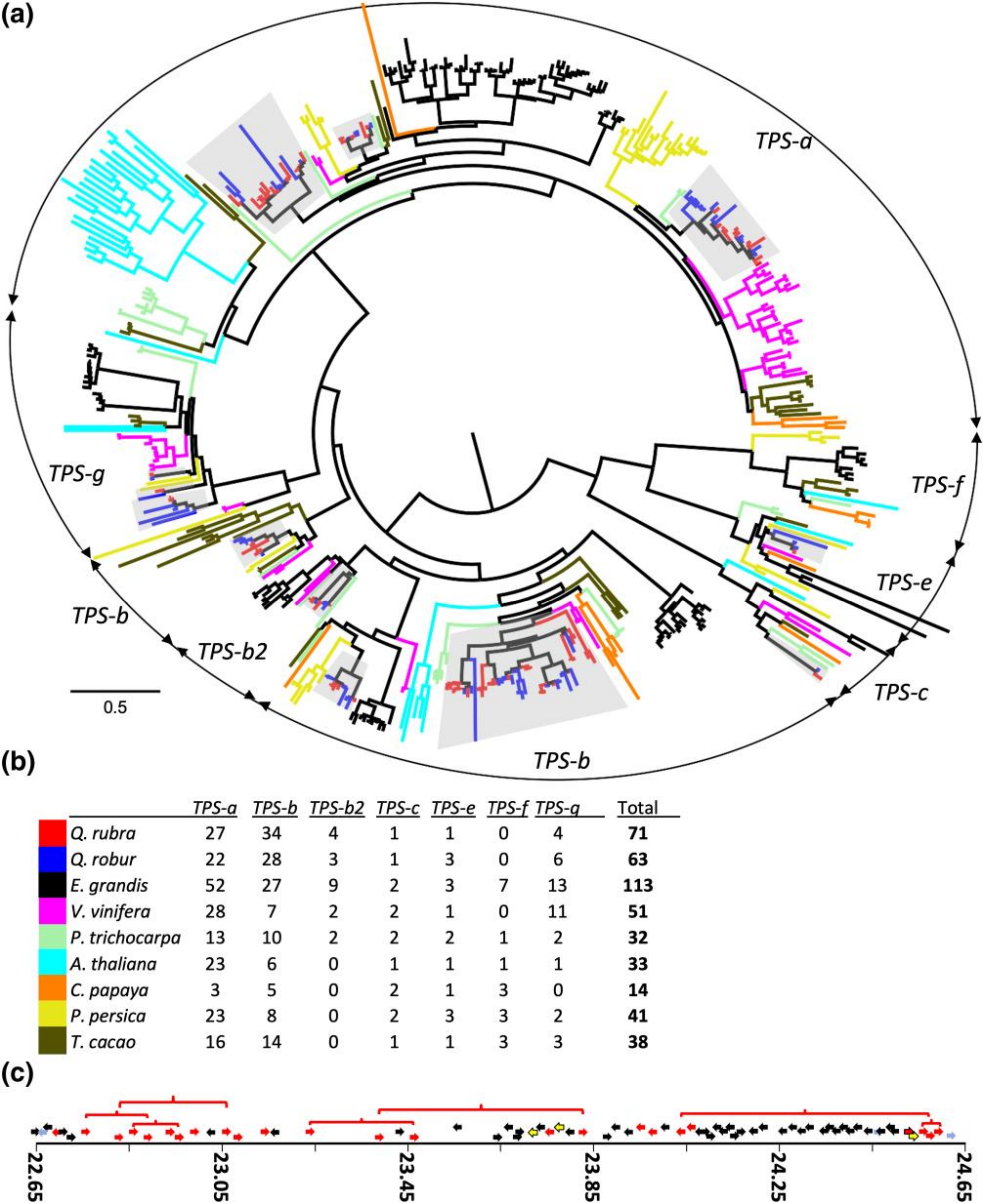
Analysis of terpene synthase genes in *Q. rubra* and comparison to 8 other plant species. a) Phylogeny of 459 TPS genes; subfamilies are shown; branch terminals are colored by species as indicated in b); *Quercus* TPS are shaded. b) Gene copy number of TPS subfamilies in *Q. rubra* and 8 other plant species. c) A TPS gene replication hotspot on chr 9 of *Q. rubra*, containing 23 putatively functional TPS-b gene models (red), 3 TPS fragments (yellow, no gene model in Phytozome) across 2,000 kb; genes putatively involved in plant secondary metabolism are shown in light blue; all other gene models in black. Red brackets show genes with high sequence homology, indicating recent gene duplication events.

Gene families of interest were examined in the *Q. rubra* assembly and 8 additional rosid species: *Q. robur*, *P. persica*, *P. trichocarpa*, *A. thaliana*, *C. papaya*, *E. grandis*, *T. cacao*, *and V. vinifera*. These species were selected due to the availability of manually curated genes from these gene families (Kulheim et al. 2015). A total of 459 genes were analyzed and classified by the TPS subfamily. Both *Q. rubra* and *Q. robur* have more TPS genes than most plant species, 71 and 63, respectively (Jiang et al. 2019). They share a gene family expansion prominent in the *TPS-b* subfamily, which mostly produces monoterpenes such as α- or β-pinene, limonene, or myrcene (Fig. 4a and b). While not all plant species have genes in the *TPS-b2* subfamily, *Q. rubra* has 4 and *Q. robur* has 3 genes. In contrast, oaks have lost the *TPS-f* subfamily, similar to *V. vinifera*. The single *TPS-c* gene in oaks likely functions as an ent-kaurene synthase, synthesizing the precursor of gibberellic acid. When comparing the *TPS-c* gene from *Q. rubra* and *Q. robur*, we can see little divergence between the 2, and they occur as a pair of orthologous genes (Fig. 4a). Overall, only 9 TPS genes are found as 1-to-1 orthologs between *Q. rubra* and *Q. robur* (Fig. 4a). Many TPS genes in subfamilies *TPS-a* and *TPS-b* occur in tandem gene arrays, originating from tandem gene duplication events due to unequal crossing-over during meiosis. In *Q. rubra*, 1 such tandem array contains 23 putative functional TPS genes as well as 3 gene fragments or pseudogenes within 2 Mb on chr 9 (Fig. 4c).

**Fig. 5.**
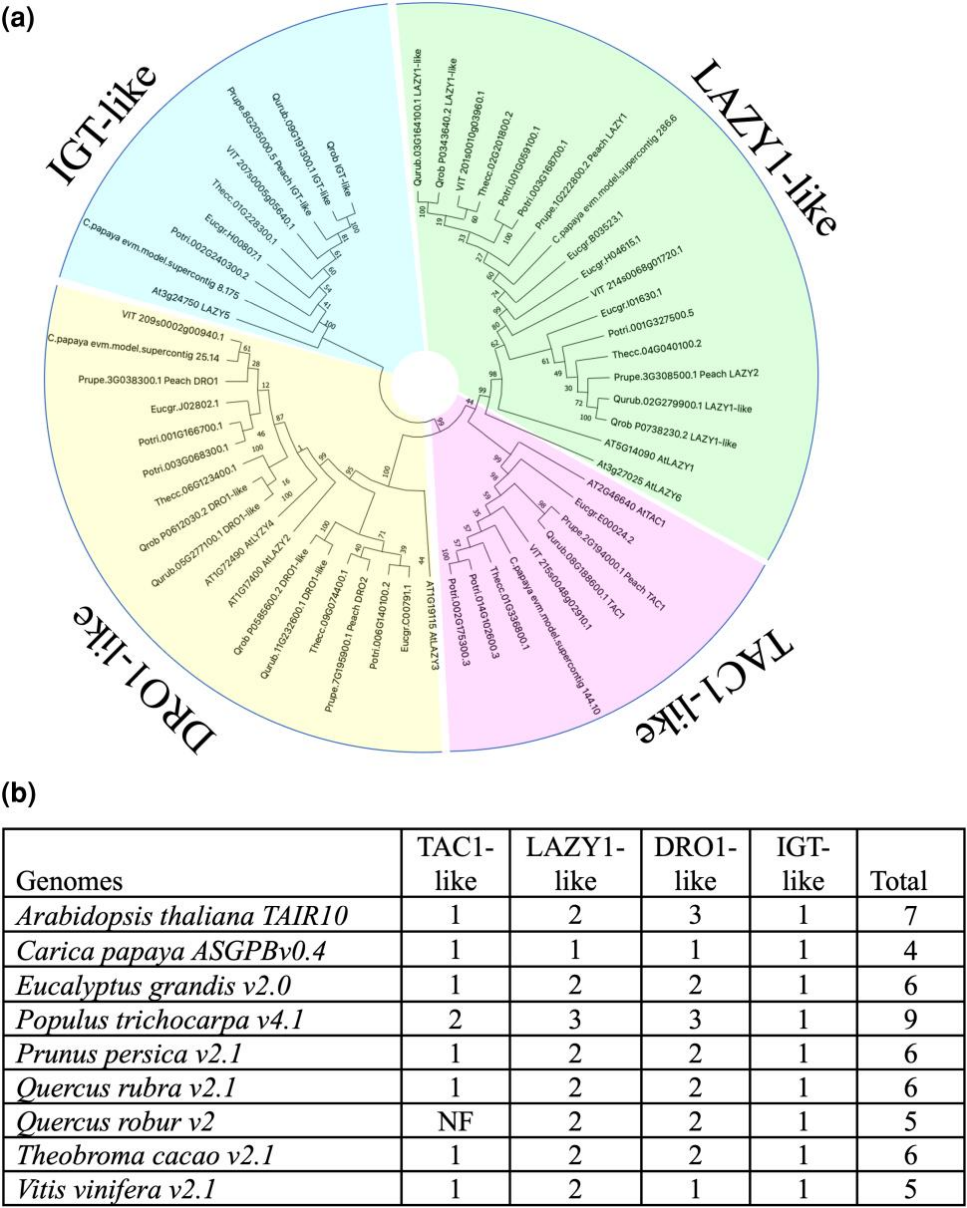
Phylogenetic analysis of the IGT gene family of *Q. rubra* and 8 other plant species. a) The maximum likelihood (ML) dendrogram shows gene family members identified in genomes of *A. thaliana*, *C. papaya*, *E. grandis*, *P. trichocarpa*, *P. persica*, *Q. rubra*, *Q. robur*, *T. cacao*, and *V. vinifera*. Bootstrap values for the clades are displayed on the dendrogram as well as gene model IDs. Four subfamilies (TAC1-like, LAZY1-like, DRO1-like, and IGT-like) were identified based on the presence of 5 conserved domains (Waite and Dardick 2021). Subclades of IGT genes highlighted as follows: TAC1-like (pink), IGT-like (green), LAZY1-like (magenta), and DRO1-like (yellow). b) Gene copy number in *Q. rubra* and 8 other plant genomes.

A small gene family, IGT, is a key controller of the architecture of trees (Waite and Dardick 2021). In the same set of 8 rosid genomes used for comparative analysis for terpene synthases, the number of IGT genes ranged from 4 in *C. papaya* to 9 in *P. trichocarpa*. Six of the IGT gene models were found in *Q. rubra*, and based on the configuration of conserved domains, they represented 4 subclades: the TAC1-, LAZY1-, DRO1-, and IGT-like proteins (Fig. 5). *Quercus robur* was similar with 5 IGT-encoded proteins but was missing a gene from the TAC1-like subfamily.

**Fig. 6.**
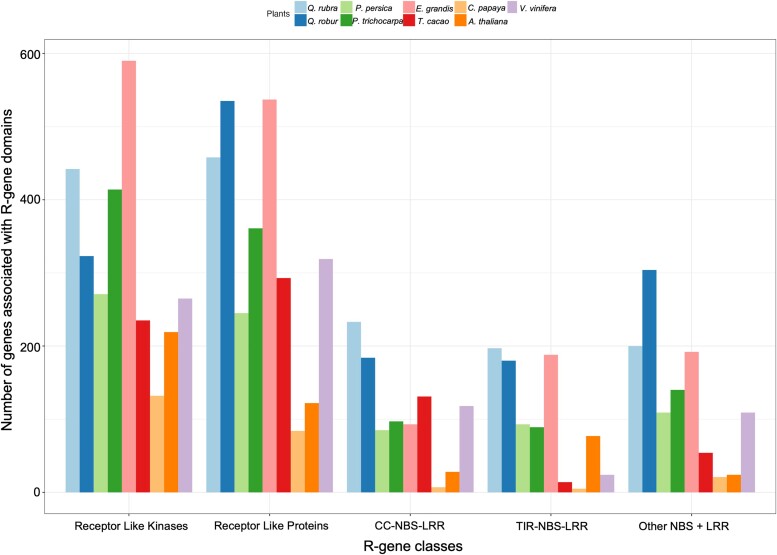
Number of genes associated with R-gene domains in *Q. rubra* and 8 other plant species (*Q. robur*, *P. persica*, *P. trichocarpa*, *E. grandis*, *T. cacao*, *C. papaya*, *A. thaliana*, and *V. vinifera*).

Plant disease resistance genes (R-genes) in *Q. rubra* and the 8 other species were identified and classified using a pipeline for genome-wide prediction of R-genes (Osuna-Cruz et al. 2018). *Quercus rubra* had 3,212 genes containing domains associated with R-genes, which was comparable to *E. grandis* (3,982), *Q. robur* (3,266), and *P. trichocarpa* (3,002). This was 3.52 times higher than in *C. papaya* (913) and 2.35 times higher than in *A. thaliana* (1,365) (Fig. 6; Supplementary Table 6 in Supplementary File 2). Genes were further categorized by the R-gene class: RLK, RLP, CNL, TNL, and other NBS-LRR. *Eucalyptus grandis* had the highest number of RLK class genes (590), with *Q. rubra* being the second highest plant species in this class (442). Out of the 9 plant species, *Q. rubra* had the highest number of CNL and TNL class genes (233 and 197, respectively) (Supplementary Table 6 in Supplementary File 2). In all species, RLK and RLP classes had more annotated genes than the NBS-LRR classes.

### LE and Mc QTLs

The timing of bud break plays an important role in the successful reproduction and continued growth of *Q. rubra* trees. Through QTL mapping, we found a single QTL for bud break timing on chr 6 spanning 6.47 Mb (30.61–37.08 Mb) (Supplementary Fig. 3a in Supplementary File 1 and Supplementary Table 7 in Supplementary File 2). The bud break region is syntenic to one end of *P. persica* chr 1 with QTLs for chill requirement and bloom date reported in a large F_2_ cross (Fan et al. 2010; Zhebentyayeva et al. 2014) and QTLs for bloom date and growing degree hours to flowering in a *P. persica* × *Prunus dulcis* cross (Supplementary Fig. 4 in Supplementary File 1) (Cantin et al. 2020). Using the QTL flanking markers, we extracted 276 genes from the bud break region on chr 6. A large number of metabolic processes have been linked to vegetative bud break, complicating the prioritization of candidate genes. GO terms associated with genes in the QTL region were linked to cellular response processes such as GINS complexes, CoA-synthesizing protein complexes, and Dom34-Hbs1 complexes. Genes were also annotated based on KEGG pathways and homology to *A. thaliana* genes (Supplementary Results 2.1). Ten genes were identified as relating to plant hormones, including 5 MYB-like DNA-binding proteins induced by hormones, 4 ethylene-responsive factors that may inhibit known flowering pathways, a putative ethylene response sensor–related protein, and a basic helix–loop–helix 104 (bHLH104) protein that is a homolog of a transcriptional repressor for glucose and abscisic acid signaling pathways (Supplementary Table 8 in Supplementary File 2). A candidate gene (*Qurub.06G162900*) present in this QTL region is a putative homolog of the circadian clock constituent cryptochrome 2 (CRY2). A gene family consisting of 4 tandemly duplicated genes in the bud break region was annotated as late embryogenesis abundant (LEA) family proteins (PFAM: PF03168). Another family in the same region with 8 tandemly duplicated genes that were annotated as serine protease inhibitor (SPI) genes encodes an inhibitor I-type family protein (PFAM: PF00280) in *Solanum tuberosum* (potato). SPIs are known to regulate proteolytic activity in plants (Clemente et al. 2019).

We found a single QTL for Mc on chr 8 of the *Q. rubra* genome spanning a 1.77 Mb region (415–2,185 kb) (Supplementary Fig. 3b in Supplementary File 1 and Supplementary Table 7 in Supplementary File 2). We extracted 81 protein-coding genes from the Mc region on chr 8. In this QTL region, a gene family having 8 tandemly duplicated genes encoding a protein previously reported as responsible for suberin deposition in the *Nicotiana benthamiana* leaves (Supplementary Table 9 in Supplementary File 2) (Martinez et al. 2015). A gene family consisting of 5 tandemly duplicated genes in this QTL region encodes a protein similar to serine protease, subtilisin, that is upregulated during senescence and expressed in the flowers of *Gladiolus grandiflorus* (Azeez et al. 2007).

### Population structure and local adaptation

**Fig. 7.**
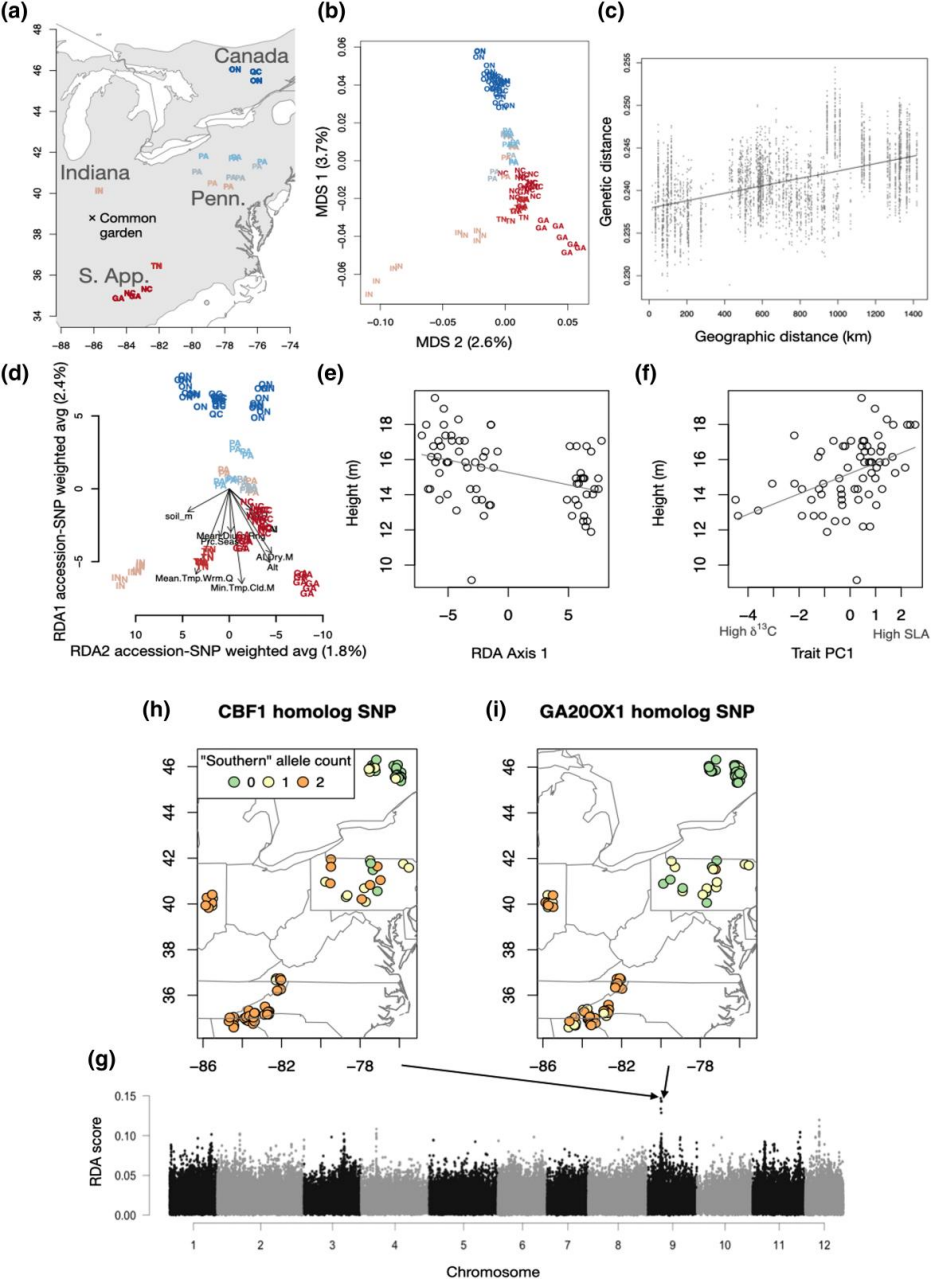
Population genetic structure and local adaptation in *Q. rubra*. a) The location of origin for trees genotyped using exome capture, combined with the common garden site (“X”) in Vallonia, Indiana. Color corresponds to latitude. b) An MDS plot of exome capture SNPs in 2 dimensions, with colors showing latitude. Labels correspond to state/province abbreviations. IN—Indiana; TN, GA, and NC—Southern Appalachians; PA—Pennsylvania; ON, and OC—Canada. c) Change in genetic distance with increasing geographic distance between pairs of genotypes, omitting those from the same provenance. d) The first 2 axes of a RDA of genome–environment associations are shown, with environmental variable loadings shown as arrows and each individual sampled tree shown as colored circles. Height in the common garden is associated with the tree’s position on the first RDA axis e) and with the first PC of trait variation f), which largely corresponds to high SLA trees (tall) vs high δ^13^C trees (short). The individual SNP loadings on RDA axes 1–2 are shown g), and the top 2 SNPs across the genome are shown h, i) with color indicating allele (orange and green—opposite homozygotes; yellow—heterozygote).

To explore range-wide population structure and patterns of selection in *Q. rubra*, targeted exome capture was performed on DNAs from 90 individuals, selected from among 76 seedlings originally sampled from a range-wide provenance trial common garden in Indiana (Coggeshall 1993) and 18 from a PA wild-collected seed nursery. The multidimensional scaling (MDS) plot showed that in 2 dimensions, population structure largely followed geographic patterns, albeit with substantial diversity within populations (e.g. Indiana trees along axis 2, Fig. 7b). Similarly, genetic distance significantly increased with geographic distance (Mantel test on a random individual from each location, *r* = 0.46, *P* = 0.0010) (Fig. 7c). However, the 2 MDS axes explained small portions of SNP variation (3.7 and 2.6%), and there was substantial genetic distance among many pairs of geographically proximate samples. Genome-wide *F*_ST_ was correspondingly low, equal to 0.0216 on average when grouping samples into 4 populations (Indiana, Southern Appalachians, PA, and Canada). Phasing of maternal and paternal chromosomes in the 18 3-member maternal half-sibling families resulted in 1,339,701 variable SNPs phased. We found that there was stronger isolation by distance among maternal chromosomes than paternal chromosomes. Geographic distance between maternal chromosomes explained 7.5% of pairwise genetic distance between unrelated individuals vs only 6.1% for paternal chromosomes (linear models), and *F*_ST_ was 0.025 for maternal chromosomes but only 0.018 for paternal chromosomes among the 3 populations that had 3-member families we phased (Indiana, Southern Appalachians, and Canada; *F*_ST_ statistics are mean of resampling subsets of unrelated individuals) (Supplementary Table 10 in Supplementary File 2).

The genome–environment RDA explained a total of 14% of the total variation in 154,076 SNPs using 9 soil and climate variables. The first RDA axis was largely temperature-associated SNP variation, and the second RDA axis was largely moisture and altitude-associated SNP variation. We also found that height and DBH in the Indiana provenance trial were both significantly lower for trees with allele frequencies mismatched to the experimental site, as estimated using distance between the predicted genotype for the provenance trial and the observed genotypes along the first 2 RDA axes (*N* = 72, height *r* = −0.305, *P* = 0.0092; DBH *r* = −0.263, *P* = 0.0258). Even more strongly, genotypes’ loading on the first RDA axis was associated with DBH and height such that the genotypes with many warm-associated alleles had greater DBH (*r* = −0.56, *P* < 10^−7^) and height (*r* = −0.40, *P* = 0.0005).

Among phenotypes, DBH was most strongly associated with stomatal density (*N* = 71, *r* = 0.31, *P* = 0.0095) and leaf C:N (*N* = 71, *r* = 0.29, *P* = 0.0153), while height was most strongly associated with the first trait principal component (PC) (*N* = 68, *r* = 0.44, *P* = 0.0002), specific leaf area (SLA) (*N* = 72, *r* = 0.43, *P* = 0.0002), and δ^13^C (*N* = 70, *r* = −0.35, *P* = 0.0033). Leaf C:N was also significantly associated with the first genomic RDA axis (*r* = −0.38, *P* = 0.0020), meaning C:N was higher for genotypes with many warm-associated alleles and lower for those with cold-associated alleles.

We scanned the genome for outlier loadings in Euclidean distance in the first 2 RDA axes. A clear peak emerged on chr 9, where the top SNP tagged a CBF1/DREB1B homolog that was among 4 potential paralogs in a tandem repeat (*Qurub.09G078300*, *Qurub.09G078400*, *Qurub.09G078500*, and *Qurub.09G078600*). The second SNP was located ∼520 kb away in a GA20OX1 homolog (*Qurub.09G081700*) and was moderately correlated in allele frequency with the top SNP (*R*^2^ = 0.54). A peak was found on chr 4 between 2 WAX2 homologs (*Qurub.04G074500* and *Qurub.04G074600*) in a region where there are 4 other WAX2 homologs. These SNPs were also significantly associated with DBH and height in the common garden, even after accounting for genome-wide similarity among individuals (*Qurub.09G078500* SNP vs height *P* = 0.004 vs DBH *P* < 10^−6^; *Qurub.09G081700* SNP vs height *P* = 0.0028 vs DBH *P* < 10^−6^; and *Qurub.04G074500* SNP vs height *P* = 0.01400 vs DBH *P* = 0.00059). A potential example of maladaptive paternal gene flow can be seen with the GA20OX1 homolog (*Qurub.09G081700*) SNP. We found 3 heterozygous individuals among the 50 we were able to phase (2 from Quebec and 1 from North Carolina), and each of these carried the locally uncommon allele (putatively maladapted) on the paternal chromosome.

### Estimation of nucleotide diversity

**Fig. 8.**
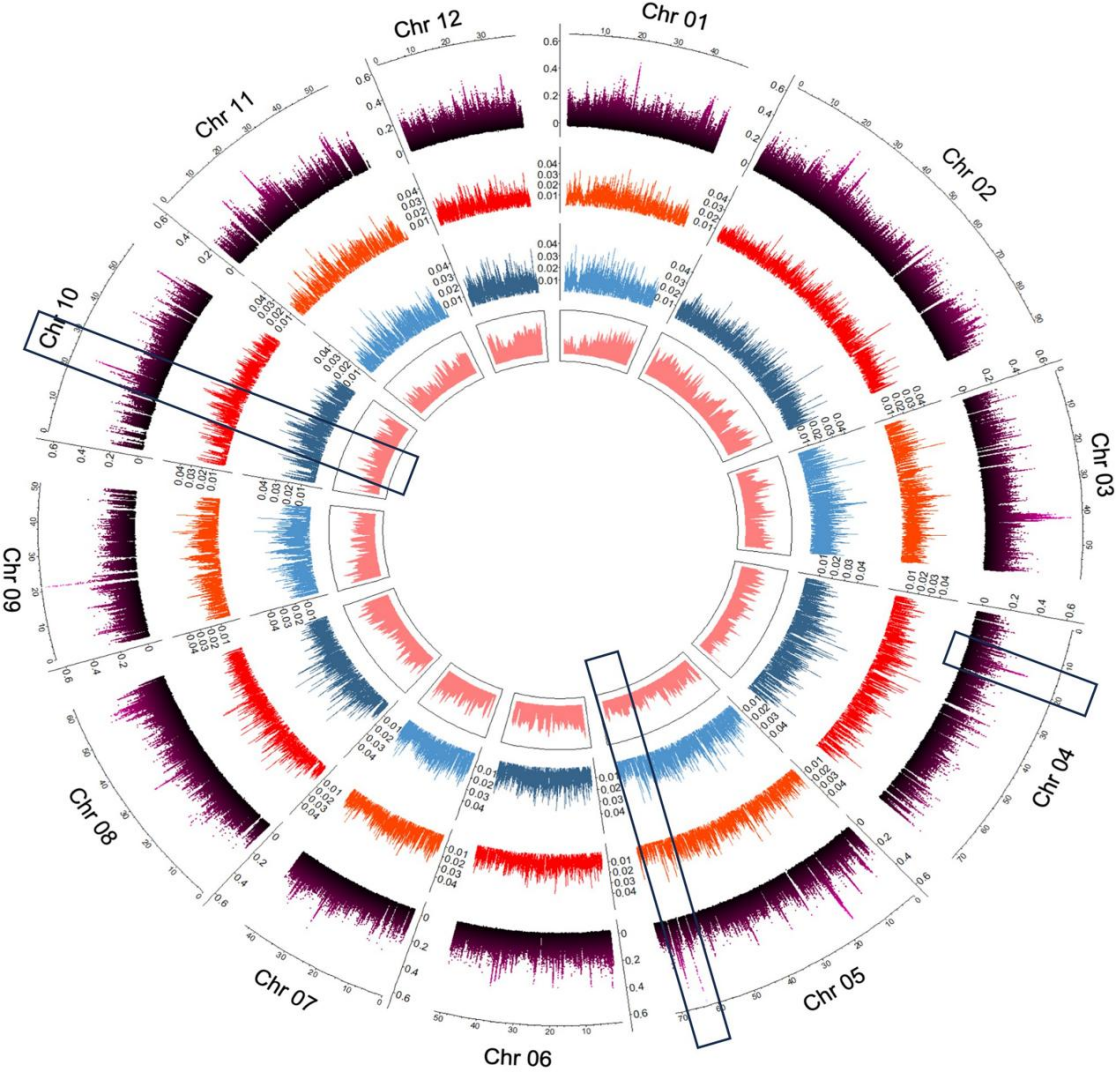
Display of genome-wide nucleotide diversity statistics and gene density from the chromosome sequences for each chromosome. A ruler with chromosome position in Mb is drawn above each chromosome. *F*_ST_ values based on SNP-by-SNP analysis are presented in purple. *π* values for the Covington population are shown in orange and for the Lisle population in blue. Boxes in the inner circle shown in pink represent gene density across each chromosome. Rectangular boxes on chr 5 and chr 10 indicate cooccurrence of *F*_ST_ peaks with nucleotide diversity and/or gene density peaks, and rectangular box on chr 4 points to *F*_ST_ peak colocation with the candidate gene from independent RDA analysis.

Whole genome sequencing of 60 *Q. rubra* adult trees split between 2 sites (Baraga Plains, MI, and Lisle, IL) yielded a mean value of genome-wide nucleotide diversity across both populations sampled of 0.008. There was no significant difference between the populations; however, the values for nucleotide diversity within each population were slightly lower, at 0.0079 in Lisle and 0.0077 in Baraga Plains, than the overall value. The values for nucleotide diversity in each population across each of the 12 chromosomes are displayed in Fig. 8. There was a significant variation in nucleotide diversity among chromosomes within each population (Supplementary Fig. 5 in Supplementary File 1). Diversity calculated in noncoding regions of the genome was 0.008, and in the coding regions, diversity was 0.0004. The overall *F*_ST_ value (0.010) showed that differentiation between these 2 populations was relatively low, showing a typical L-shaped distribution of values ranging from 0 to 0.66 across each of the chromosomes (Supplementary Fig. 6 in Supplementary File 1). Some *F*_ST_ peaks appear to cooccur with nucleotide diversity and gene density peaks within chromosomes (e.g. chr 10, Fig. 8), and 1 *F*_ST_ peak colocalized with the WAX2 homologs on chr 4 identified from the RDA above.

## Discussion

### High-quality genome assembly

The genus *Quercus* contains approximately half of the species in the Fagaceae family. Genomic resources have recently been added in the section *Quercus* with a recently published reference genome for *Q. lobata*, *Q. mongolica*, and *Quercus variabilis* (Ai et al. 2022; Han et al. 2022; Sork et al. 2022). We produced the first high-quality, annotated reference genome in the Lobatae section, a diploid, haplotype-phased, chromosome-level genome for *Q. rubra*. According to the short-read alignment statistics, BUSCO scores, and comparisons with other oak genomes, this *Q. rubra* genome is largely complete and accurately scaffolded and annotated. Over 3,000 well-supported SVs were found in *Q. rubra* by mapping long reads to the genome, providing a set of SVs that could be driving phenotypic diversity in this tree species. Orienting and numbering the chromosomes to the previously published *Q. rubra* linkage map (Konar et al. 2017) provide a foundation for further research including trait mapping in the 3-generation family. For the purposes of future comparative genomics, *Q. rubra* chromosomes are numbered consistently between *Q. rubra* and other white oak section genomes, including *Q. robur*, *Q. lobata*, and *Q. mongolica.* However, chromosomes 1, 5, 6, 10, and 11 are in reversed orientation in the *Q. rubra* genome relative to the others (Supplementary Fig. 2 in Supplementary File 1). Even with PacBio long reads and Hi-C data, current assembly pipelines are incapable of generating a fully phased haplotype-resolved assembly without progeny or parental information (Michael and VanBuren 2020; Zhou et al. 2020). Postassembly phasing of the *Q. rubra* genome yielded 1,730 haploblocks with an average length of ∼400 kb. New technologies, such as 10x Genomics linked-read sequencing or progeny sequencing, in a breeding program are still required to accurately define and track full haplotype blocks (Yu et al. 2021).

### Comparative genomic analysis

Comparison of *Q. rubra* with *Q. lobata* and *Q. mongolica* showed high collinearity between the 3 oak species (Fig. 3). The average identity of *Q. lobata* and *Q. mongolica* was 95.36% whereas the average identity of *Q. rubra* genome to *Q. lobata* and *Q. mongolica* was 91.65 and 91.78%, respectively. As members of the section *Quercus*, we expected *Q. lobata* and *Q. mongolica* to be more similar to each other than to *Q. rubra*. Also, the presence of shared SVs between these 3 genomes suggests that rearrangements are not an assembly error.

Many plant genomes contain a large number of repetitive sequences, which mostly consist of transposable elements. Estimates of repetitive content from 2 previously published *Quercus* genomes, 54.00% for *Q. lobata* and 53.75% for *Q. mongolica* (Ai et al. 2022; Sork et al. 2022), are similar to the 50.15% found in *Q. rubra*. LTR elements, such as *Copia* and *Gypsy*, were the most common repetitive elements in all 3 sequenced genomes, and long interspersed repeats accounted for the majority of non-LTR elements.

### Terpene synthase gene family analysis

Terpenes play diverse and important roles in plants from phytohormones such as gibberellic acid to photosynthetic pigments (carotenes and the phytol tail of chlorophyll) to interactions with the abiotic and biotic environment. *Eucalyptus grandis* has been noted as having the largest number of TPS genes, 113 total, found among sequenced plant genomes (Kulheim et al. 2015). The 2 *Quercus* species (*Q. rubra* and *Q. robur*) had the second most TPS genes (71 and 63) of the 9 species examined but were closer in number to *V. vinifera* with 51 and *P. persica* with 41 TPS genes. The tandem array of 23 TPS genes from *Q. rubra* is larger than the largest tandem array in *E. grandis*, which has 10 putative functional TPS and 7 TPS pseudogenes in 400 kb on chr 6.

Much of the “smoke” in the Great Smoky Mountains National Park (USA) is volatile organic compounds emitted by oaks and dominated by isoprene and monoterpenes. TPS genes in subfamily *TPS-b2* typically produce the monoterpene (*E*)-β-ocimene or the hemiterpene isoprene. Not all plant species have the ability to produce isoprene or have genes in this subfamily; however, *Q. rubra* has 4 of these genes and *Q. robur* has 3. There are 4 amino acids required for a *TPS-b2* to produce isoprene (Sharkey et al. 2013). None of the 7 oak *TPS-b2* has all 4 amino acids described as necessary for isoprene production; however, *Qurub.08G134300* and *Qurob.P0198710* have 3 out of 4 and are the most likely candidate genes for isoprene production.

### IGT gene family comparison

The TAC1- and LAZY1-like clades have opposing influence on axillary shoot growth angle (Hollender et al. 2020; Waite and Dardick 2021). *Quercus rubra* has 1 functional gene of the TAC1 subfamily on chr 8 and 2 functional genes for LAZY1 on chrs 2 and 3, potentially contributing to tree architecture.

Genes from the DRO1-like clade named after DEEPER ROOTING 1 (DRO1) from rice have severe impact on root branching pattern and directional growth of lateral roots’ relative gravity force direction and surface of the soil (Guseman et al. 2017; Uga et al. 2013). Two DRO1-like genes on chrs 5 and 11 were identified in the *Q. rubra* genome (Fig. 5). The *Qurub.11G232600* gene on chr 11 has an intact exon–intron structure and is expressed, while a second family member, *Qurub.05G277100* gene, encodes putatively nonfunctional proteins with missing domains I, II, and V. The intact DRO1-like gene is a potential candidate for control of the deep-rooting tendency of *Q. rubra* (Waite and Dardick 2021), which is an important drought avoidance strategy in this species not shared across all oaks.

### Disease resistance (R-genes) gene family analysis

Some of the recent oak genome studies have suggested that the expansion of disease resistance gene families is critical for the viability of long-lived plants (Plomion et al. 2018; Sork et al. 2022); however, others do not support this hypothesis (Ai et al. 2022). Plomion et al. (2018) found that there are 1,091 NBS-LRR genes present in *Q. robur*, and Sork et al. (2022) report 751 strong R-gene candidates and 2,176 possible R-genes in the *Q. lobata* genome. The number of genes associated with R-gene domains in *Q. rubra* is comparable to *Q. robur* and *Q. lobata*. However, Ai et al. (2022) identified 1,215 R-genes in *Q. mongolica*, which is considerably lower than in the *Q. rubra* genome (3,212). Previous research has demonstrated that in a reduced disease pressure environment, most plants can lower the cost of resistance by eliminating disease resistance genes (Grant et al. 1998; Stahl et al. 1999), suggesting that the maintenance of a high number of R-genes in *Q. rubra*, *Q. robur*, and *Q. lobata* could be due to high pressure from pests and pathogens.

### Diversity and adaptation

Genetic and nucleotide diversity assessments among populations of *Q. rubra* were conducted to illuminate opportunities for investigating sources of variation associated with local adaptation with genome-wide investigations enabled by the reference genome.

#### Population genetics and adaptation

MDS plots and *F*_ST_ values calculated for 4 sampled geographic population clusters (Southern Appalachian, Indiana, PA, and Eastern Canada) showed that population genetic differentiation largely followed geographic distances, but most diversity is found within populations, e.g. there was substantial genetic distance among many geographically proximate samples. Observations of high genetic variation, or allele richness, within populations accompanied by low genetic differentiation among populations have often been reported in forests trees, including *Quercus* species, employing morphological or neutral genetic markers (as reviewed by Backs and Ashley 2021; Porth and El-Kassaby 2014). Our finding of greater geographic structure for maternal chromosomes than paternal chromosomes may be due to the larger distance traveled by *Quercus* wind-dispersed pollen as opposed to vertebrate-dispersed seeds. This pattern has been observed earlier using small numbers of markers genotyped on dissected maternal tissues (Iwaizumi et al. 2010; Mimura and Aitken 2007) but less documented across the whole genome.

Oaks are model species for investigating the basis of adaptive genetic variation in forest trees, especially with the advent of genomic resources (Backs and Ashley 2021; Lazic et al. 2021). We implemented RDA to characterize multilocus associations of SNP variation with 9 potentially important environmental variables (Brun et al. 2022). Our hypothesis that a genome–environment RDA could capture genomic signatures for local adaptation to climate was supported by the fact that the RDA-predicted mismatch of each genotype for the common garden site predicted fitness proxies of DBH and height. This site was fairly warm (with hotter summers than the Southern Appalachian sites), and indeed, the genotype loadings on the first RDA axis were also associated with height and DBH. Specifically, genotypes with warm-associated alleles showed greater DBH and height, potentially suggesting an acquisitive life history strategy with faster growth associated with warm temperature–associated alleles.

Trait–size associations also suggested physiological strategies associated with growth in the common garden, with large DBH being strongly associated with high stomatal density and leaf C:N and height associated with the first trait PC that indicated especially high SLA and low δ^13^C for taller trees. Furthermore, leaf C:N was also significantly associated with the first genomic RDA axis (*r* = −0.37), meaning C:N was higher for genotypes with multiple warm-associated alleles and lower for those with cold-associated alleles. With the exception of C:N, these trait associations suggest that resource capture strategies are characteristic of large trees with warm-adapted alleles in the southern Indiana common garden.

A peak of RDA outlier climate-associated loci identified on chr 9 was located in a tandem array of CBF1/DREB1B genes, which in *A. thaliana* play a key role in regulating cold acclimation (Thomashow 1999) and dehydration response (Liu et al. 1998). A moderately correlated SNP was also found in a GA20OX1 homolog (*Qurub.09G0817have*), which was previously reported to elevate bioactive GA and cause a loss of normal growth cessation under short days when the *A. thaliana* GA20OX1 gene was overexpressed in *P. trichocarpa* (Eriksson et al. 2015). Interestingly, Zhou et al. (2017) reported growth retardation in *A. thaliana* in response to low temperature caused by CBF1/DREB genes was accompanied by repression of GA biosynthesis due to the accumulation of DELLA proteins. Thus, CBF1/DREB1B and GA20OX1 homolog SNPs may be of interest for targeted marker-assisted selection in tree improvement programs. Finally, an RDA outlier region was found on chr 4 between 2 WAX2 homologs (*Qurub.04G074500* and *Qurub.04G074600*) in a region with 4 other WAX2 homologs. As this locus controls cuticular wax production in *A. thaliana* (Chen et al. 2021) and *Lithocarpus* spp. (Yang et al. 2018), cuticular waxes may also play an important role in local adaptation in *Q. rubra*.

#### Nucleotide diversity

The low mean values of genome-wide nucleotide diversity (*π*) within and among the 2 populations sampled near Lisle, IL, and Baraga Plains, MI, are not unexpected given the relatively close geographic positions of populations (∼600 km), relative to the wide range of *Q. rubra* across eastern North America, and previous estimates for genome-wide nucleotide diversity in oaks. Overall genome-wide nucleotide diversity in our study for *Q. rubra* was only slightly lower compared with the *π* values calculated based on the whole genome resequencing of 20 individuals from 1 population of *Q. robur* (0.01) (Plomion et al. 2018) and those from 639 individuals from 4 age-structured cohorts of *Quercus petraea* (0.0012) (Saleh et al. 2022). In a recent study, Nocchi et al. (2022) resequenced the whole genome of 360 *Q. robur* individuals from 4 British parkland sites and reported *π* values of 0.007, which is consistent with the values we detected. Interestingly, both nucleotide diversity values and the *F*_ST_ values for genetic diversity (population differentiation) varied noticeably across each of the individual chromosomes. The cooccurrence of peaks in *F*_ST_, nucleotide diversity, and gene density values (e.g. chr 5 and chr 10, Fig. 8) and colocation of a high *F*_ST_ peak on chr 4 with the *Qurub.04G074500* WAX2 homolog candidate gene from the independent RDA analysis indicate that these genome regions should be of interest in further studies of adaptive evolution in oaks.

### Conclusion

A high-quality, chromosome-scale, haplotype-resolved genome of 739.58 Mb (over 95% of the 1C genome) was assembled for *Q. rubra*, an ecologically and economically important oak species. This genome is also the first representative of the Lobatae section of genus *Quercus*. The reference tree was selected from the F_2_ generation of a genetic mapping family, which provides a resource for the association of phenotypes with the 33,333 protein-coding genes identified. Initial studies with the *Q. rubra* reference genome provide insights into variations in chromosome structure within the oak clade and important adaptive traits and pathways, including disease resistance, terpene synthesis, vegetative bud break, Mc, and physiological strategies associated with growth and stress response.

## Supplementary Material

jkad209_Supplementary_Data

## Data Availability

The *Q. rubra* genome assembly and annotation are available at https://phytozome-next.jgi.doe.gov/info/Qrubra_v2_1 (Phytozome genome ID: 687). Raw sequences have been submitted to Sequence Read Archive at NCBI (BioProject accessions—PRJNA938173 and PRJNA973109; SRA accessions—Illumina short reads: SRR23696575, PacBio long reads: SRR23696574, and Hi-C data: SRR23696573). Supplemental material available at G3 online.

## Data citations

[Illumina DNA sequences for *Q. rubra* genome] Kapoor B., Jenkins J., Schmutz J., Zhebentyayeva T., Kuelheim C., Coggeshall M., Heim C., Lasky J.R., Leites L., Islam-Faridi N., Romero-Severson J., DeLeo V.L., Lazic D., Gailing O., Carlson J.E., Staton M.; 2023; Haplotype resolved chromosome-level genome assembly of Northern red oak (*Quercus rubra* L.), NCBI Sequence Read Archive (SRA); PRJNA938173; SRR23696575.

[PacBio long reads for *Q. rubra* genome] Kapoor B., Jenkins J., Schmutz J., Zhebentyayeva T., Kuelheim C., Coggeshall M., Heim C., Lasky J.R., Leites L., Islam-Faridi N., Romero-Severson J., DeLeo V.L., Lazic D., Gailing O., Carlson J.E., Staton M.; 2023; Haplotype resolved chromosome-level genome assembly of Northern red oak (*Quercus rubra* L.), NCBI Sequence Read Archive (SRA); PRJNA938173; SRR23696574.

[Hi-C data for *Q. rubra* genome] Kapoor B., Jenkins J., Schmutz J., Zhebentyayeva T., Kuelheim C., Coggeshall M., Heim C., Lasky J.R., Leites L., Islam-Faridi N., Romero-Severson J., DeLeo V.L., Lazic D., Gailing O., Carlson J.E., Staton M.; 2023; Haplotype resolved chromosome-level genome assembly of Northern red oak (*Quercus rubra* L.), NCBI Sequence Read Archive (SRA); PRJNA938173; SRR23696573.

[Illumina RNA-seq reads for *Q. rubra* annotation] Hardwood Genomics Team; 2015; Northern Red Oak transcriptome sequencing from a variety of adult tissues and abiotic stress-exposed seedlings; NCBI Sequence Read Archive (SRA); PRJNA273270.

[*Quercus rubra* genome sequence] Kapoor B., Jenkins J., Schmutz J., Zhebentyayeva T., Kuelheim C., Coggeshall M., Heim C., Lasky J.R., Leites L., Islam-Faridi N., Romero-Severson J., DeLeo V.L., Lazic D., Gailing O., Carlson J.E., Staton M.; *Quercus rubra* v2.1, Phytozome; Phytozome genome ID: 687; https://phytozome-next.jgi.doe.gov/info/Qrubra_v2_1.

[*Quercus rubra* genome annotation] Kapoor B., Jenkins J., Schmutz J., Zhebentyayeva T., Kuelheim C., Coggeshall M., Heim C., Lasky J.R., Leites L., Islam-Faridi N., Romero-Severson J., DeLeo V.L., Lazic D., Gailing O., Carlson J.E., Staton M.; *Quercus rubra* v2.1, Phytozome; Phytozome genome ID: 687; https://phytozome-next.jgi.doe.gov/info/Qrubra_v2_1.

[*Quercus rubra* genome files] Kapoor B., Jenkins J., Schmutz J., Zhebentyayeva T., Kuelheim C., Coggeshall M., Heim C., Lasky J.R., Leites L., Islam-Faridi N., Romero-Severson J., DeLeo V.L., Lazic D., Gailing O., Carlson J.E., Staton M.; *Quercus rubra* v2.1, Phytozome; Phytozome genome ID: 687; https://phytozome-next.jgi.doe.gov/info/Qrubra_v2_1.

## References

[jkad209-B1] Abadía A , Gil E, Morales F, Montañés L, Montserrat G, Abadía J. 1996. Marcescence and senescence in a submediterranean oak (*Quercus subpyrenaica* E.H. del Villar): photosynthetic characteristics and nutrient composition. Plant Cell Environ. 19(6):685–694. doi:10.1111/j.1365-3040.1996.tb00403.x.

[jkad209-B2] Abbott AG , Zhebentyayeva T, Barakat A, Liu Z, Plomion C, Adam-Blondon A-F, Plomion C, Adam-Blondon A-F. 2015. The genetic control of bud-break in trees. In: Plomion C, Adam-Blondon A-F, editors. Advances in Botanical Research. vol. 74. Amsterdam, Netherlands: Elsevier. p. 201–228.

[jkad209-B3] Ai W , Liu Y, Mei M, Zhang X, Tan E, Liu H, Han X, Zhan H, Lu X. 2022. A chromosome-scale genome assembly of the Mongolian oak (*Quercus mongolica*). Mol Ecol Resour. 22(6):2396–2410. doi:10.1111/1755-0998.13616. 35377556

[jkad209-B4] Alexander LW , Woeste KE. 2014. Pyrosequencing of the northern red oak (*Quercus rubra* L.) chloroplast genome reveals high quality polymorphisms for population management. Tree Genet Genomes. 10(4):803–812. doi:10.1007/s11295-013-0681-1.

[jkad209-B5] Altschul SF , Gish W, Miller W, Myers EW, Lipman DJ. 1990. Basic local alignment search tool. J Mol Biol. 215(3):403–410. doi:10.1016/S0022-2836(05)80360-2. 2231712

[jkad209-B6] Azeez A , Sane AP, Bhatnagar D, Nath P. 2007. Enhanced expression of serine proteases during floral senescence in Gladiolus. Phytochemistry 68(10):1352–1357. doi:10.1016/j.phytochem.2007.02.027. 17412375

[jkad209-B7] Backs JR , Ashley MV. 2021. Quercus genetics: insights into the past, present, and future of oaks. Forests 12(12):1628. doi:10.3390/f12121628.

[jkad209-B8] Bisgrove R . 2010. Urban horticulture: future scenarios. Acta Hortic. 881(881):33–46. doi:10.17660/ActaHortic.2010.881.1.

[jkad209-B9] Brun P , Zimmermann NE, Hari C, Pellissier L, Karger DN. 2022. Global climate-related predictors at kilometer resolution for the past and future. Earth Syst Sci Data. 14(12):5573–5603. doi:10.5194/essd-14-5573-2022.

[jkad209-B10] Cantin CM , Wang X-W, Almira M, Arús P, Eduardo I. 2020. Inheritance and QTL analysis of chilling and heat requirements for flowering in an interspecific almond x peach (Texas x Earlygold) F2 population. Euphytica 216(3):51. doi:10.1007/s10681-020-02588-9.

[jkad209-B11] Chen Y , Zhang L, Zhang H, Chen L, Yu D. 2021. ERF1 delays flowering through direct inhibition of FLOWERING LOCUS T expression in Arabidopsis. J Integr Plant Biol. 63(10):1712–1723. doi:10.1111/jipb.13144. 34152677

[jkad209-B12] Clemente M , Corigliano MG, Pariani SA, Sánchez-López EF, Sander VA, Ramos-Duarte VA. 2019. Plant serine protease inhibitors: biotechnology application in agriculture and molecular farming. Int J Mol Sci. 20(6):E1345. doi:10.3390/ijms20061345. PMC6471620 30884891

[jkad209-B13] Coggeshall M . 1993. Oak tree improvement in Indiana. Annales Des Sci Forestières. 50(Supplement):416s–419s. doi:10.1051/forest:19930748.

[jkad209-B14] Dold C , Thomas AL, Ashworth AJ, Philipp D, Brauer DK, Sauer TJ. 2019. Carbon sequestration and nitrogen uptake in a temperate silvopasture system. Nutr. Cycl. Agroecosystems. 114:85–98. doi:10.1007/s10705-019-09987-y.

[jkad209-B15] Dougherty L , Borejsza-Wysocka E, Miaule A, Wang P, Zheng D, Jansen M, Brown S, Piñeros M, Dardick C, Xu K. 2023. A single amino acid substitution in MdLAZY1A dominantly impairs shoot gravitropism in Malus. bioRxiv 535771. doi:10.1101/2023.04.05.535771, preprint: not peer reviewed. 37394917

[jkad209-B16] Dudley JW , Moll RH. 1969. Interpretation and use of estimates of heritability and genetic variances in plant breeding1. Crop Sci. 9(3):257–262. doi:10.2135/cropsci1969.0011183X000900030001x.

[jkad209-B17] Dutt M , Mahmoud LM, Nehela Y, Grosser JW, Killiny N. 2022. The *Citrus sinensis* TILLER ANGLE CONTROL 1 (CsTAC1) gene regulates tree architecture in sweet oranges by modulating the endogenous hormone content. Plant Sci. 323:111401. doi:10.1016/j.plantsci.2022.111401. 35905898

[jkad209-B18] Ehleringer JR , Werk KS. 1986. Modifications of solar-radiation absorption patterns and implications for carbon gain at the leaf level. In: Givnish T, editors. On the Economy of Plant Form and Function. Cambridge: Cambridge University Press. p. 57–82.

[jkad209-B19] Ellwood ER , Temple SA, Primack RB, Bradley NL, Davis CC. 2013. Record-breaking early flowering in the eastern United States. PLoS One 8(1):e53788. doi:10.1371/journal.pone.0053788. 23342001 PMC3547064

[jkad209-B20] Emms DM , Kelly S. 2019. Orthofinder: phylogenetic orthology inference for comparative genomics. Genome Biol. 20(1):1–14. doi:10.1186/s13059-019-1832-y. 31727128 PMC6857279

[jkad209-B21] Eriksson ME , Hoffman D, Kaduk M, Mauriat M, Moritz T. 2015. Transgenic hybrid aspen trees with increased gibberellin (GA) concentrations suggest that GA acts in parallel with FLOWERING LOCUS T2 to control shoot elongation. New Phytol. 205(3):1288–1295. doi:10.1111/nph.13144. 25382585

[jkad209-B22] Ezcurra E , Montana C, Arizaga S. 1991. Architecture, light interception, and distribution of *Larrea* species in the Monte Desert, Argentina. Ecology 72(1):23–34. doi:10.2307/1938899.

[jkad209-B23] Falavigna VS , Guitton B, Costes E, Andrés F. 2019. I want to (bud) break free: the potential role of *DAM* and *SVP*-like genes in regulating dormancy cycle in temperate fruit trees. Front Plant Sci. 9:1990. doi:10.3389/fpls.2018.01990. 30687377 PMC6335348

[jkad209-B24] Fan S , Bielenberg DG, Zhebentyayeva TN, Reighard GL, Okie WR, Holland D, Abbott AG. 2010. Mapping quantitative trait loci associated with chilling requirement, heat requirement and bloom date in peach (*Prunus persica*). New Phytol. 185(4):917–930. doi:10.1111/j.1469-8137.2009.03119.x. 20028471

[jkad209-B25] Fasoula VA , Fasoula DA. 2002. Principles underlying genetic improvement for high and stable crop yield potential. Field Crops Res. 75(2–3):191–209. doi:10.1016/S0378-4290(02)00026-6.

[jkad209-B26] Fladung M . 2021. Targeted CRISPR/Cas9-based knock-out of the rice orthologs *TILLER ANGLE CONTROL 1 (TAC1*) in poplar induces erect leaf habit and shoot growth. Forests 12(12):1615. doi:10.3390/f12121615.

[jkad209-B27] Forester BR , Lasky JR, Wagner HH, Urban DL. 2018. Comparing methods for detecting multilocus adaptation with multivariate genotype–environment associations. Mol Ecol. 27(9):2215–2233. doi:10.1111/mec.14584. 29633402

[jkad209-B28] Glenn DM , Bassett CB, Tworkoski T, Scorza R, Miller SS. 2015. Tree architecture of pillar and standard peach affect canopy transpiration and water use efficiency. Sci Hortic. 187:30–34. doi:10.1016/j.scienta.2015.02.030.

[jkad209-B200] Goodstein DM , Shu S, Howson R, Neupane R, Hayes RD, Fazo J, Mitros T, Dirks W, Hellsten U, Putnam N, Rokhsar DS. 2012. Phytozome: a comparative platform for green plant genomics. Nucleic Acids Res. 40(D1):D1178–86. doi:10.1093/nar/gkr944. 22110026 PMC3245001

[jkad209-B29] Goel M , Sun H, Jiao W-B, Schneeberger K. 2019. SyRI: finding genomic rearrangements and local sequence differences from whole-genome assemblies. Genome Biol. 20(1):1–13. doi:10.1186/s13059-019-1911-0. 31842948 PMC6913012

[jkad209-B30] Goudet J . 2005. Hierfstat, a package for r to compute and test hierarchical F-statistics. Mol Ecol Notes. 5(1):184–186. doi:10.1111/j.1471-8286.2004.00828.x.

[jkad209-B31] Grant MR , McDowell JM, Sharpe AG, de Torres Zabala M, Lydiate DJ, Dangl JL. 1998. Independent deletions of a pathogen-resistance gene in *Brassica* and *Arabidopsis*. Proc Natl Acad Sci USA. 95(26):15843–15848. doi:10.1073/pnas.95.26.15843. 9861058 PMC28132

[jkad209-B32] Grímsson F , Grimm GW, Zetter R, Denk T. 2016. Cretaceous and Paleogene Fagaceae from North America and Greenland: evidence for a Late Cretaceous split between *Fagus* and the remaining Fagaceae. Acta Palaeobotanica 56(2):247–305. doi:10.1515/acpa-2016-0016.

[jkad209-B33] Gugger PF , Peñaloza-Ramírez JM, Wright JW, Sork VL. 2017. Whole-transcriptome response to water stress in a California endemic oak, *Quercus lobata*. Tree Physiol. 37(5):632–644. doi:10.1093/treephys/tpw122. 28008082

[jkad209-B34] Guseman JM , Webb K, Srinivasan C, Dardick C. 2017. DRO1 influences root system architecture in *Arabidopsis* and *Prunus* species. Plant J. 89(6):1093–1105. doi:10.1111/tpj.13470. 28029738

[jkad209-B35] Han B , Longxin W, Xian Y, Xie X-M, Li W-Q, Zhao Y, Zhang R-G, Qin X, Li D-Z, Jia K-H. 2022. A chromosome-level genome assembly of the Chinese cork oak (*Quercus variabilis*). Front Plant Sci. 13:1001583. doi:10.3389/fpls.2022.1001583. 36212310 PMC9538376

[jkad209-B36] Hart AJ , Ginzburg S, Xu MS, Fisher CR, Rahmatpour N, Mitton JB, Paul R, Wegrzyn JL. 2020. EnTAP: bringing faster and smarter functional annotation to non-model eukaryotic transcriptomes. Mol Ecol Resour. 20(2):591–604. doi:10.1111/1755-0998.13106. 31628884

[jkad209-B37] Heberling JM , Muzika R-M. 2023. Not all temperate deciduous trees are leafless in winter: the curious case of marcescence. Ecosphere 14(3):e4410. doi:10.1002/ecs2.4410.

[jkad209-B38] Heim C . 2016. Quantitative Trait Loci Mapping and Analysis of Adaptive Traits in Northern Red Oak (Quercus rubra L.). Columbia (MO): University of Missouri-Columbia.

[jkad209-B39] Hill JL , Hollender CA. 2019. Branching out: new insights into the genetic regulation of shoot architecture in trees. Curr Opin Plant Biol. 47:73–80. doi:10.1016/j.pbi.2018.09.010. 30339931

[jkad209-B40] Hollender CA , Hill JL, Waite J, Dardick C. 2020. Opposing influences of *TAC1* and *LAZY1* on lateral shoot orientation in Arabidopsis. Sci Rep. 10(1):6051. doi:10.1038/s41598-020-62962-4. 32269265 PMC7142156

[jkad209-B41] Iwaizumi MG , Takahashi M, Watanabe A, Ubukata M. 2010. Simultaneous evaluation of paternal and maternal immigrant gene flow and the implications for the overall genetic composition of *Pinus densiflora* dispersed seeds. J Heredity. 101(2):144–153. doi:10.1093/jhered/esp089. 19889720

[jkad209-B42] Jiang S-Y , Jin J, Sarojam R, Ramachandran S. 2019. A comprehensive survey on the terpene synthase gene family provides new insight into its evolutionary patterns. Genome Biol Evol. 11(8):2078–2098. doi:10.1093/gbe/evz142. 31304957 PMC6681836

[jkad209-B43] Konar A , Choudhury O, Bullis R, Fiedler L, Kruser JM, Stephens MT, Gailing O, Schlarbaum S, Coggeshall MV, Staton ME, et al 2017. High-quality genetic mapping with ddRADseq in the non-model tree *Quercus rubra*. BMC Genomics 18(1):417. doi:10.1186/s12864-017-3765-8. 28558688 PMC5450186

[jkad209-B44] Krzywinski M , Schein J, Birol İ, Connors J, Gascoyne R, Horsman D, Jones SJ, Marra MA. 2009. Circos: an information aesthetic for comparative genomics. Genome Res. 19(9):1639–1645. doi:10.1101/gr.092759.109. 19541911 PMC2752132

[jkad209-B45] Kubisiak TL , Nelson CD, Staton ME, Zhebentyayeva T, Smith C, Olukolu BA, Fang G-C, Hebard FV, Anagnostakis S, Wheeler N, et al 2013. A transcriptome-based genetic map of Chinese chestnut (*Castanea mollissima*) and identification of regions of segmental homology with peach (*Prunus persica*). Tree Genet Genomes. 9(2):557–571. doi:10.1007/s11295-012-0579-3.

[jkad209-B46] Kulheim C , Padovan A, Hefer C, Krause ST, Köllner TG, Myburg AA, Degenhardt J, Foley WJ. 2015. The Eucalyptus terpene synthase gene family. BMC Genomics 16(1):450. doi:10.1186/s12864-015-1598-x. 26062733 PMC4464248

[jkad209-B47] Lagesen K , Hallin P, Rødland EA, Staerfeldt H-H, Rognes T, Ussery DW. 2007. RNAmmer: consistent and rapid annotation of ribosomal RNA genes. Nucleic Acids Res. 35(9):3100–3108. doi:10.1093/nar/gkm160. 17452365 PMC1888812

[jkad209-B48] Lazic D , Hipp AL, Carlson JE, Gailing O. 2021. Use of genomic resources to assess adaptive divergence and introgression in oaks. Forests 12(6):690. doi:10.3390/f12060690.

[jkad209-B49] Leites LP , Rehfeldt GE, Steiner KC. 2019. Adaptation to climate in five eastern North America broadleaf deciduous species: growth clines and evidence of the growth-cold tolerance trade-off. Perspect Plant Ecol Evol Syst. 37:64–72. doi:10.1016/j.ppees.2019.02.002.

[jkad209-B50] Le Provost G , Lesur I, Lalanne C, Da Silva C, Labadie K, Aury JM, Leple JC, Plomion C. 2016. Implication of the suberin pathway in adaptation to waterlogging and hypertrophied lenticels formation in pedunculate oak (*Quercus robur* L.). Tree Physiol. 36(11):1330–1342. doi:10.1093/treephys/tpw056. 27358207

[jkad209-B51] Lind-Riehl J , Gailing O. 2015. Fine-scale spatial genetic structure of two red oak species, *Quercus rubra* and *Quercus ellipsoidalis*. Plant Syst Evol. 301(6):1601–1612. doi:10.1007/s00606-014-1173-y.

[jkad209-B52] Little EL . 1979. Checklist of United States Trees (Native and Naturalized). DC: Forest Service, US Department of Agriculture.

[jkad209-B53] Liu Q , Kasuga M, Sakuma Y, Abe H, Miura S, Yamaguchi-Shinozaki K, Shinozaki K. 1998. Two transcription factors, DREB1 and DREB2, with an EREBP/AP2 DNA binding domain separate two cellular signal transduction pathways in drought- and low-temperature-responsive gene expression, respectively, in Arabidopsis. Plant Cell 10(8):1391–1406. doi:10.1105/tpc.10.8.1391. 9707537 PMC144379

[jkad209-B54] Maere S , Heymans K, Kuiper M. 2005. BiNGO: a Cytoscape plugin to assess overrepresentation of Gene Ontology categories in Biological Networks. Bioinformatics 21(16):3448–3449. doi:10.1093/bioinformatics/bti551. 15972284

[jkad209-B55] Magalhães AP , Verde N, Reis F, Martins I, Costa D, Lino-Neto T, Castro PH, Tavares RM, Azevedo H. 2016. RNA-Seq and gene network analysis uncover activation of an ABA-dependent signalosome during the cork oak root response to drought. Front Plant Sci. 6:1195. doi:10.3389/fpls.2015.01195. 26793200 PMC4707443

[jkad209-B56] Makela M , Michael P, Theriault G, Nkongolo KK. 2016. High genetic variation among closely related red oak (*Quercus rubra*) populations in an ecosystem under metal stress: analysis of gene regulation. Genes Genomics. 38(10):967–976. doi:10.1007/s13258-016-0441-3.

[jkad209-B57] Marçais G , Delcher AL, Phillippy AM, Coston R, Salzberg SL, Zimin A. 2018. MUMmer4: a fast and versatile genome alignment system. PLoS Comput Biol. 14(1):e1005944. doi:10.1371/journal.pcbi.1005944. 29373581 PMC5802927

[jkad209-B58] Martin M , Patterson M, Garg S, Fischer S, Pisanti N, Klau GW, Schöenhuth A, Marschall T. 2016. Whatshap: fast and accurate read-based phasing. BioRxiv 085050. doi:10.1101/085050, preprint: not peer reviewed.

[jkad209-B59] Martinez DE , Borniego ML, Battchikova N, Aro E-M, Tyystjärvi E, Guiamét JJ. 2015. SASP, a senescence-associated subtilisin protease, is involved in reproductive development and determination of silique number in *Arabidopsis*. J Exp Bot. 66(1):161–174. doi:10.1093/jxb/eru409. 25371504

[jkad209-B60] Mendes FK , Vanderpool D, Fulton B, Hahn MW. 2020. CAFE 5 models variation in evolutionary rates among gene families. Bioinformatics. 36(22-23):5516. 10.1093/bioinformatics/btaa1022. 33325502

[jkad209-B61] Michael TP , VanBuren R. 2020. Building near-complete plant genomes. Curr Opin Plant Biol. 54:26–33. doi:10.1016/j.pbi.2019.12.009. 31981929

[jkad209-B62] Millers I , Shriner DS, Rizzo D. 1989. History of Hardwood Decline in the Eastern United States. vol. 126. Washington (DC): US Department of Agriculture, Forest Service, Northeastern Forest Experiment.

[jkad209-B63] Mimura M , Aitken SN. 2007. Adaptive gradients and isolation-by-distance with postglacial migration in *Picea sitchensis*. Heredity (Edinb). 99(2):224–232. doi:10.1038/sj.hdy.6800987. 17487214

[jkad209-B64] Murray SC , Rooney WL, Mitchell SE, Sharma A, Klein PE, Mullet JE, Kresovich S. 2008. Genetic improvement of sorghum as a biofuel feedstock: II. QTL for stem and leaf structural carbohydrates. Crop Sci. 48(6):2180–2193. doi:10.2135/cropsci2008.01.0068.

[jkad209-B65] Neale DB , Kremer A. 2011. Forest tree genomics: growing resources and applications. Nat Rev Genet. 12(2):111–122. doi:10.1038/nrg2931. 21245829

[jkad209-B66] Nesom G . 2001. Northern Red Oak (*Quercus rubra* L.).

[jkad209-B67] Nocchi G , Brown N, Coker TLR, Plumb WJ, Stocks JJ, Denman S, Buggs RJA. 2022. Genomic structure and diversity of oak populations in British parklands. Plants People Planet 4(2):167–181. doi:10.1002/ppp3.10229.

[jkad209-B68] Ontl TA , Janowiak MK, Swanston CW, Daley J, Handler S, Cornett M, Hagenbuch S, Handrick C, Mccarthy L, Patch N. 2020. Forest management for carbon sequestration and climate adaptation. Journal of Forestry. 118(1):86–101.

[jkad209-B69] Osuna-Cruz CM , Paytuvi-Gallart A, Di Donato A, Sundesha V, Andolfo G, Aiese Cigliano R, Sanseverino W, Ercolano MR. 2018. PRGdb 3.0: a comprehensive platform for prediction and analysis of plant disease resistance genes. Nucleic Acids Res. 46(D1):D1197–D1201. doi:10.1093/nar/gkx1119. 29156057 PMC5753367

[jkad209-B70] Ou S , Chen J, Jiang N. 2018. Assessing genome assembly quality using the LTR Assembly Index (LAI). Nucleic Acids Res. 46(21):e126. doi:10.1093/nar/gky730. 30107434 PMC6265445

[jkad209-B71] Oyama K , Herrera-Arroyo ML, Rocha-Ramírez V, Benítez-Malvido J, Ruiz-Sánchez E, González-Rodríguez A. 2017. Gene flow interruption in a recently human-modified landscape: the value of isolated trees for the maintenance of genetic diversity in a Mexican endemic red oak. For Ecol Manage. 390:27–35. doi:10.1016/j.foreco.2017.01.018.

[jkad209-B72] Plomion C , Aury J-M, Amselem J, Leroy T, Murat F, Duplessis S, Faye S, Francillonne N, Labadie K, Le Provost G, et al 2018. Oak genome reveals facets of long lifespan. Nat Plants. 4(7):440–452. doi:10.1038/s41477-018-0172-3. 29915331 PMC6086335

[jkad209-B73] Porth I , El-Kassaby YA. 2014. Assessment of the genetic diversity in forest tree populations using molecular markers. Diversity (Basel) 6(2):283–295. doi:10.3390/d6020283.

[jkad209-B74] Purcell S , Neale B, Todd-Brown K, Thomas L, Ferreira MAR, Bender D, Maller J, Sklar P, de Bakker PIW, Daly MJ, et al 2007. PLINK: a tool set for whole-genome association and population-based linkage analyses. Am J Hum Genet. 81(3):559–575. doi:10.1086/519795. 17701901 PMC1950838

[jkad209-B75] Rauschendorfer J , Rooney R, Külheim C. 2022. Strategies to mitigate shifts in red oak (*Quercus* sect. *Lobatae*) distribution under a changing climate. Tree Physiol. 42(12):2383–2400. doi:10.1093/treephys/tpac090. 35867476

[jkad209-B76] Rodríguez-Correa H , Oyama K, Quesada M, Fuchs EJ, González-Rodríguez A. 2018. Contrasting patterns of population history and seed-mediated gene flow in two endemic Costa Rican oak species. J Heredity. 109(5):530–542. doi:10.1093/jhered/esy011. 29509902

[jkad209-B77] Saleh D , Chen J, Leplé J, Leroy T, Truffaut L, Dencausse B, Lalanne C, Labadie K, Lesur I, Bert D, et al 2022. Genome-wide evolutionary response of European oaks during the Anthropocene. Evol Lett. 6(1):4–20. doi:10.1002/evl3.269. 35127134 PMC8802238

[jkad209-B78] Sander IL . 1990. *Quercus rubra* L. overcup oak. Silvics North Am. 2:727–733.

[jkad209-B79] Schwartz MD , Ahas R, Aasa A. 2006. Onset of spring starting earlier across the Northern Hemisphere. Glob Chang Biol. 12(2):343–351. doi:10.1111/j.1365-2486.2005.01097.x.

[jkad209-B80] Scotti-Saintagne C , Mariette S, Porth I, Goicoechea PG, Barreneche T, Bodénès C, Burg K, Kremer A. 2004. Genome scanning for interspecific differentiation between two closely related oak species [*Quercus robur* L. and *Q. petraea* (Matt.) Liebl.]. Genetics 168(3):1615–1626. doi:10.1534/genetics.104.026849. 15579711 PMC1448783

[jkad209-B81] Sedlazeck FJ , Rescheneder P, Smolka M, Fang H, Nattestad M, von Haeseler A, Schatz MC. 2018. Accurate detection of complex structural variations using single-molecule sequencing. Nat Methods. 15(6):461–468. doi:10.1038/s41592-018-0001-7. 29713083 PMC5990442

[jkad209-B82] Seppey M , Manni M, Zdobnov EM. 2019. Gene Prediction. New York (NY): Springer. p. 227–245.

[jkad209-B83] Sharkey TD , Gray DW, Pell HK, Breneman SR, Topper L. 2013. Isoprene synthase genes form a monophyletic clade of acyclic terpene synthases in the TPS-B terpene synthase family. Evolution 67(4):1026–1040. doi:10.1111/evo.12013. 23550753

[jkad209-B84] Singh RK , Maurya JP, Azeez A, Miskolczi P, Tylewicz S, Stojkovič K, Delhomme N, Busov V, Bhalerao RP. 2018. A genetic network mediating the control of bud break in hybrid aspen. Nat Commun. 9(1):4173. doi:10.1038/s41467-018-06696-y. 30301891 PMC6177393

[jkad209-B85] Soltani N , Best T, Grace D, Nelms C, Shumaker K, Romero-Severson J, Moses D, Schuster S, Staton M, Carlson J, et al 2020. Transcriptome profiles of *Quercus rubra* responding to increased O3 stress. BMC Genomics 21(1):160. doi:10.1186/s12864-020-6549-5. 32059640 PMC7023784

[jkad209-B86] Sork VL , Bramble J, Sexton O. 1993. Ecology of mast-fruiting in three species of North American deciduous oaks. Ecology 74(2):528–541. doi:10.2307/1939313.

[jkad209-B87] Sork VL , Cokus SJ, Fitz-Gibbon ST, Zimin AV, Puiu D, Garcia JA, Gugger PF, Henriquez CL, Zhen Y, Lohmueller KE, et al 2022. High-quality genome and methylomes illustrate features underlying evolutionary success of oaks. Nat Commun. 13(1):2047. doi:10.1038/s41467-022-29584-y. 35440538 PMC9018854

[jkad209-B88] Stahl EA , Dwyer G, Mauricio R, Kreitman M, Bergelson J. 1999. Dynamics of disease resistance polymorphism at the *Rpm1* locus of *Arabidopsis*. Nature 400(6745):667–671. doi:10.1038/23260. 10458161

[jkad209-B89] Stavi I , Thevs N, Welp M, Zdruli P. 2022. Provisioning ecosystem services related with oak (Quercus) systems: a review of challenges and opportunities. Agroforestry Systems. 1–21.

[jkad209-B90] Thomashow MF . 1999. Plant cold acclimation: freezing tolerance genes and regulatory mechanisms. Annu Rev Plant Physiol Plant Mol Biol. 50(1):571–599. doi:10.1146/annurev.arplant.50.1.571. 15012220

[jkad209-B91] Uga Y , Sugimoto K, Ogawa S, Rane J, Ishitani M, Hara N, Kitomi Y, Inukai Y, Ono K, Kanno N, et al 2013. Control of root system architecture by DEEPER ROOTING 1 increases rice yield under drought conditions. Nat Genet. 45(9):1097–1102. doi:10.1038/ng.2725. 23913002

[jkad209-B92] van Ooijen JW , Boer MP, Jansen R, Maliepaard C. 2000. MapQTL 4.0: Software for the Calculation of QTL Positions on Genetic Maps. Wageningen, Netherlands: Plant Research International.

[jkad209-B93] Vengadesan G , Pijut PM. 2009. In vitro propagation of northern red oak (*Quercus rubra* L.). Vitro Cell Dev Biol Plant. 45(4):474–482. doi:10.1007/s11627-008-9182-6.

[jkad209-B94] Verde I , Abbott AG, Scalabrin S, Jung S, Shu S, Marroni F, Zhebentyayeva T, Dettori MT, Grimwood J, Cattonaro F, et al 2013. The high-quality draft genome of peach (*Prunus persica*) identifies unique patterns of genetic diversity, domestication and genome evolution. Nat Genet. 45(5):487–494. doi:10.1038/ng.2586. 23525075

[jkad209-B95] Vila-Viçosa C , Gonçalves J, Honrado J, Lomba Â, Almeida RS, Vázquez FM, Garcia C. 2020. Late Quaternary range shifts of marcescent oaks unveil the dynamics of a major biogeographic transition in southern Europe. Sci Rep. 10(1):21598. doi:10.1038/s41598-020-78576-9. 33298997 PMC7726089

[jkad209-B96] Waite JM , Dardick C. 2021. The roles of the IGT gene family in plant architecture: past, present, and future. Curr Opin Plant Biol. 59:101983. doi:10.1016/j.pbi.2020.101983. 33422965

[jkad209-B97] Wang Z , Cao H, Zhang C, Chen F, Liu Y. 2022. The SNF5-type protein BUSHY regulates seed germination via the gibberellin pathway and is dependent on HUB1 in Arabidopsis. Planta 255(2):34. doi:10.1007/s00425-021-03767-1. 35006338

[jkad209-B98] Yang C-K , Huang B-H, Ho S-W, Huang M-Y, Wang J-C, Gao J, Liao P-C. 2018. Molecular genetic and biochemical evidence for adaptive evolution of leaf abaxial epicuticular wax crystals in the genus *Lithocarpus* (Fagaceae). BMC Plant Biol. 18(1):196. doi:10.1186/s12870-018-1420-4. 30223774 PMC6142356

[jkad209-B99] Yu J , Hulse-Kemp AM, Babiker E, Staton M. 2021. High-quality reference genome and annotation aids understanding of berry development for evergreen blueberry (*Vaccinium darrowii*). Hortic Res. 8(1):228. doi:10.1038/s41438-021-00641-9. 34719668 PMC8558335

[jkad209-B100] Zhebentyayeva TN , Fan S, Chandra A, Bielenberg DG, Reighard GL, Okie WR, Abbott AG. 2014. Dissection of chilling requirement and bloom date QTLs in peach using a whole genome sequencing of sibling trees from an F2 mapping population. Tree Genet Genomes. 10(1):35–51. doi:10.1007/s11295-013-0660-6.

[jkad209-B101] Zhou M , Chen H, Wei D, Ma H, Lin J. 2017. *Arabidopsis* CBF3 and DELLAs positively regulate each other in response to low temperature. Sci Rep. 7(1):39819. doi:10.1038/srep39819. 28051152 PMC5209670

[jkad209-B102] Zhou Q , Tang D, Huang W, Yang Z, Zhang Y, Hamilton JP, Visser RGF, Bachem CWB, Robin Buell C, Zhang Z, et al 2020. Haplotype-resolved genome analyses of a heterozygous diploid potato. Nat Genet. 52(10):1018–1023. doi:10.1038/s41588-020-0699-x. 32989320 PMC7527274

