# Supplementary material for: A haplotype-resolved chromosome-scale genome for Quercus rubra L. provides insights into the genetics of adaptive traits for red oak species
Source: G3 (Bethesda). 2023 Sep 14;13(11):jkad209. doi: 10.1093/g3journal/jkad209 (PMC10627279; doi:10.1093/g3journal/jkad209)
Supplement: jkad209_Supplementary_Data [file jkad209_supplementary_data.zip › File_S1_G3-2023-404308.docx]

A haplotype-resolved chromosome-scale genome for *Quercus rubra* L. provides insights into the genetics of adaptive traits for red oak species

– Supplemental Methods, Results and Figures

Beant Kapoor^1^, Jerry Jenkins^2^, Jeremy Schmutz^2^, Tatyana Zhebentyayeva^3,8^, Carsten Kuelheim^4^, Mark Coggeshall^5^, Chris Heim^6^, Jesse R. Lasky^7^, Laura Leites^8^, Nurul Islam-Faridi^9^, Jeanne Romero-Severson^10^, Victoria L. DeLeo^7^, Sarah M. Lucas^7^, Desanka Lazic^11^, Oliver Gailing^11^, John Carlson^8^, Margaret Staton^1^

^1^Department of Entomology and Plant Pathology, University of Tennessee, Knoxville, TN 37996, U.S.A

^2^HudsonAlpha Institute for Biotechnology, Huntsville, AL 35806, U.S.A

^3^Department of Forestry and Natural Resources, University of Kentucky, Lexington, KY 40506, U.S.A

^4^College of Forest Resources and Environmental Science, Michigan Tech University, Houghton, MI 49931, U.S.A

^5^College of Agriculture, Food and Natural Resources, University of Missouri, Columbia, MO 65211, U.S.A

^6^Horticultural Science, North Carolina State University, Raleigh, NC 27695, U.S.A

^7^Department of Biology, Pennsylvania State University, University Park, PA 16802, U.S.A

^8^Department of Ecosystem Science and Management, Pennsylvania State University, University Park, PA 16802, U.S.A

^9^Forest Tree Molecular Cytogenetics Laboratory, USDA-FS, SRS-4160; Department of Ecology & Conservation Biology, Texas A&M University, College Station, TX 77843, U.S.A

^10^Department of Biological Sciences, University of Notre Dame, Notre Dame, IN 46556, U.S.A

^11^Department of Forest Genetics and Forest Tree Breeding, University of Göttingen, Göttingen, Lower Saxony, 37077 Germany

**Corresponding author**: Margaret Staton; E-mail: [mstaton1@utk.edu](mailto:mstaton1@utk.edu)

**Table of contents**

[**1. Supplementary Methods 3**](#_omjzkwb6k1jr)

1.1. Plant materials 3

1.2. High molecular weight genomic DNA extraction 3

1.3. DNA sequencing 3

[1.4. Reference genome assembly 4](#_ea1nbncqdfue)

[1.5. Genome annotation 4](#_oo8yr0xh07vl)

[1.6. Fluorescence in situ hybridization (FISH) with rDNA oligonucleotide probes 5](#_ihxhdgnk9aeg)

[1.7. Comparative genomics analyses 6](#_vuqssxtpbib3)

[1.8. Gene family comparisons 7](#_h315ffhg5u1v)

[1.9. Synteny between P. persica v2.1 and Q. rubra v2.1 genomes 8](#_3s88xn780yoe)

1.10. Population structure and local adaptation 8

1.11. Estimation of nucleotide diversity 9

[**2. Supplementary Results 10**](#_w3tmqxs77b7s)

[2.1. Candidate genes within leaf emergence QTL interval 10](#_jkpfch8p3zs)

[2.1.1. Plant hormones (8 genes) 10](#_emijca5gggzu)

[2.1.2. Chromatin remodelling (9 genes) 10](#_duilausmoqcj)

[2.1.3. Transcriptional factors – stimuli responsive (3 genes) 11](#_xe04z6kzru7c)

[2.1.4. Light sensing -Photoperiod receptor- circadian clocks 11](#_4r1htacgbveb)

[**3. Supplemental Figures 12**](#_cevfdv99j2jb)

# Supplementary Methods

## Plant materials

A *Q. rubra* tree from a three-generation pedigree (West Lafayette, IN, U.S.A) was selected for the reference genome sequence. The first generation of the pedigree were parent trees SM1 and SM2 on the campus of Purdue University from which a full-sib mapping population (pseudo-F_2_) and a dense genetic map were generated [(Konar et al., 2017)](https://www.zotero.org/google-docs/?5EM3UG). Two second-generation (F_1_) individuals (SM1316 and SM1370) were crossed to create a third generation. The high-quality reference genome was generated from accession #2932, one of the eight third-generation trees produced. This F_2_ progeny tree was chosen as reference to provide a unique, three-generation pedigree for genome-enabled inheritance studies, with the expectation of increased homozygosity to enhance genome assembly.

## High molecular weight genomic DNA extraction

For Illumina and PacBio sequencing, approx. 10 g each of dormant leaf bud and twig bark tissues were collected from the F_2_ reference tree. For Oxford Nanopore sequencing, approx. 20 g of leaves were collected and shipped to Penn State University (State College, PA, U.S.A) for high molecular weight DNA extraction. Genomic DNA was extracted from frozen tissues using a modified CTAB protocol as described in [Callahan et al., 2021](https://www.zotero.org/google-docs/?Z1Lhq9) with final high-salt clean-up from polysaccharides following guidelines provided by PacBio [(Pacific Biosciences, 2014)](https://www.zotero.org/google-docs/?j5ekjE). DNA samples were quantified for high molecular weight using a Qubit^®^ 2.0 fluorometer (Invitrogen, Carlsbad, CA, U.S.A) and intactness was checked on 0.8% agarose gels. Final quality control (QC) of the genomic DNA before PacBio and Nanopore sequencing employed ScreenTape DNA Analysis (Agilent Technologies). For Hi-C sequencing, young leaf tissues were collected from the F_2_ reference tree as they emerged soon after bud break in May 2020. The tissues were flash-frozen in liquid nitrogen immediately after collection and shipped at liquid nitrogen temperature to the HudsonAlpha Biotechnology Institute (HAI) (Huntsville, AL, U.S.A) for Hi-C library preparation.

## DNA sequencing

A TruSeq DNA PCR-Free library was constructed with genomic DNA from the F_2_ *Q. rubra* tree. The library was subjected to 2x150 paired-end sequencing on an Illumina NextSeq 550 mid-output instrument by the Penn State University Genomics Center, producing approx. 26 Gb of high-quality DNA sequence data, representing approx. 33x genome coverage.

High molecular weight genomic DNA from the F_2_ *Q. rubra* reference tree was sheared and then subjected to size selection, retaining all fragments above 15 Kb size. A SMRTbell Express (PacBio) library was prepared from the size selected DNA, and sequenced by the Penn State University Genomics Core Facility. They produced approx. 50x depth of DNA sequence (approx. N50 read length of 20 Kb) with one SMRT cell run on a PacBio Sequel (Pacific Biosciences, Inc.) machine. The HAI produced approx. 563 million 2×150 bp PE150 sequence reads on an Illumina NovaSeq 6000 S4 instrument from the F_2_ reference tree genome for use in Hi-C DNA chromatin conformation capture, as per the modified protocol of [Ma et al., 2021](https://www.zotero.org/google-docs/?og7laj).

## Reference genome assembly

The primary *Q. rubra* genome assembly (genome hereafter) was generated with MECAT (Xiao et al., 2017) using 50.60x PacBio long sequence coverage (average read length of 11,447 bp), and the resulting assembly was then polished using RACON [(Vaser et al., 2017)](https://www.zotero.org/google-docs/?e9P7qE). Using Hi-C Illumina data, 142 misjoins were identified in the polished assembly. Scaffolds were then manually ordered, oriented, and joined together using Hi-C sequence data [(Ma et al., 2021)](https://www.zotero.org/google-docs/?C3YmNH). Contigs terminating in significant telomeric sequence were properly oriented in the assembly. A total of 861 joins were applied to 873 contigs to form the final assembly consisting of 12 chromosomes. Chromosomes were oriented and numbered using the publicly available *Q. rubra* linkage map [(Konar et al., 2017)](https://www.zotero.org/google-docs/?KNMgRC). Adjacent alternative haplotypes were identified on the joined contigs. Alternative haplotype regions were then collapsed using the longest common substring between the two haplotypes. A total of 234 adjacent alternative haplotypes were resolved. We corrected heterozygous Single Nucleotide Polymorphism (SNP) and Insertions and Deletions (INDEL) phasing errors using the PacBio long sequence data. A total of 22,891 (0.36% of the 6,375,469) heterozygous SNPs and INDELs were corrected. Additionally, we corrected homozygous SNPs and INDELs using 29x Illumina read coverage (2x150, 400 bp insert). We then used WhatsHap, a weighted haplotype assembler that uses both statistical information and coverage depth, to assign reads to parental haplotypes based on Single Nucleotide Variants (SNVs) found in PacBio long-read data [(Martin et al., 2016)](https://www.zotero.org/google-docs/?jOaHKg).

To validate the correspondence of the 12 longest *Q. rubra* assembly chromosomes, we used an existing linkage map consisting of 957 sequence markers (mostly SNPs) [(Konar et al., 2017)](https://www.zotero.org/google-docs/?KyYvGg). We aligned marker sequences to our assembly using BLASTN 2.12.0+ [(Altschul et al., 1990)](https://www.zotero.org/google-docs/?Pm4wt7). All hits had a nucleotide identity of more than 80% and an e-value of ≤ 8.94e-16.

To characterize structural variants (SVs), the PacBio reads were aligned to the *Q. rubra* genome using minimap2 v2.24-r1122 in map-pb mode [(Li, 2018)](https://www.zotero.org/google-docs/?78h55P). The output was converted to bam using Samtools v1.16, which was then provided to Sniffles v2.0.7 to call variants with lengths of 10 Kb or more [(Danecek et al., 2011; Sedlazeck et al., 2018)](https://www.zotero.org/google-docs/?qZZS22). The resulting vcf file was filtered using Bcftools v1.16 to keep only heterozygous variants of length 1 Mb or less, and depth of at least five PacBio reads supporting the reference and alternate haplotypes, respectively [(Danecek et al., 2011)](https://www.zotero.org/google-docs/?ozZQZP). A subset of variants was visualized in Integrated Genomics Viewer v2.15.2 (IGV) to confirm the accuracy of the breakpoint sites [(Robinson et al., 2011)](https://www.zotero.org/google-docs/?S2ueTL).

## Genome annotation

PERTRAN was used to generate transcript assemblies using 2x101 bp stranded, paired-end Illumina RNA-seq reads (BioProject: PRJNA273270), which performs genome-guided transcriptome short read assembly via Genomic Short-read Nucleotide Alignment Program (GSNAP) and builds splice alignment graphs after alignment validation, realignment, and correction [(Soltani et al., 2020; Wu & Nacu, 2010)](https://www.zotero.org/google-docs/?psDU63). 144,245 transcript assemblies were constructed using the Program to Assemble Spliced Alignments (PASA) from RNA-seq transcript assemblies [(Haas et al., 2003)](https://www.zotero.org/google-docs/?oyTKZu). Repetitive regions in the genome were soft-masked using RepeatMasker [(Smit et al., 2015)](https://www.zotero.org/google-docs/?Q5Jj8J) with up to 2 Kb extension on both ends unless extending into another locus on the same strand. Repeat libraries consisting of *de novo* repeats were generated by RepeatModeler [(Flynn et al., 2020)](https://www.zotero.org/google-docs/?iVHaZ5) from the *Q. rubra* genome and repeats in RepBase. Gene loci were determined by transcript assembly alignments and/or EXONERATE alignments of proteins from *Arabidopsis thaliana*, *Glycine max*, *Populus trichocarpa*, *Theobroma cacao*, *Vitis vinifera*, *Prunus persica*, *Oryza sativa*, and *Brachypodium distachyon* [(Boeckmann et al., 2003)](https://www.zotero.org/google-docs/?2bksgR). Gene models were predicted by homology-based predictors, FGENESH+ [(Salamov & Solovyev, 2000)](https://www.zotero.org/google-docs/?FSCTEf), FGENESH_EST, and EXONERATE [(Slater & Birney, 2005)](https://www.zotero.org/google-docs/?zAfQZv), and PASA assembly open reading frames (ORFs). The best predictions for each locus were chosen based on a combination of positive factors such as expressed sequence tags (EST) and protein support, as well as one negative factor, overlap with repeats. PASA improved the selected gene predictions, adding untranslated regions (UTRs), splicing correction, and alternative transcripts. To obtain Cscore and protein coverage, PASA-improved gene model proteins were subjected to protein homology analysis to the previously mentioned proteomes. Protein coverage is the highest percentage of protein sequence alignment to the closest matching homologs in the NCBI database, and Cscore is a protein BlastP score ratio to mutual best hit (MBH) BlastP score. Cscore, protein coverage, EST coverage, and coding sequences (CDS) overlapping with repeats were used to select PASA-improved transcripts. The transcripts were chosen if their Cscore was greater than 0.5 and their protein coverage was greater than 0.5, but their CDS overlapping with repeats was less than 20%. For gene models whose CDS overlaps with repeats of more than 20%, their Cscore must be at least 0.9 and homology coverage at least 70% to be selected. The selected gene models were subject to Pfam analysis and gene models whose protein overlapped more than 30% with Pfam transposable elements (TE) domains were removed. Incomplete gene models, low homology gene models and short single exons (<300 bp CDS) without protein domains or sufficient gene expression models were manually filtered out. Additionally, we used RNAmmer v1.2 to characterize the location of the 5S, 18S, and 28S rDNA subunits in the *Q. rubra* genome [(Lagesen et al., 2007)](https://www.zotero.org/google-docs/?ohQsOE).

## Fluorescence in situ hybridization (FISH) with rDNA oligonucleotide probes

Actively growing root tips approximately 1 cm in length were harvested from young, greenhouse-grown *Q. rubra* seedlings and immediately pre-treated with 2.5 mM (~0.036%, w/v) 8-hydroxyquiloine for 4 h in the dark at room temperature (RT). The root tips were then rinsed briefly with ddH_2_O and fixed in 4:1 (95% ethanol:glacial acetic acid) and stored at RT. Finally, the fixed root tips were then enzymatically digested to prepare the chromosome spreads without cover-glass squashes as described in [Jewell & Islam-Faridi, 1994](https://www.zotero.org/google-docs/?KLdTpt), barring a modification of the enzyme mixture as described below. Fixed root tips were rinsed by swirling in ddH_2_O to wash 3X at 15 min each (to remove the fixative), mildly hydrolyzed (0.02 N HCl) at 60^o^ C for 10 min and another 10 min at RT, followed by 2X washes in ddH_2_O at RT for 10 min each. Finally, root tips were rinsed with 0.01 M ice-cold citrate buffer (pH 4.5) and washed 2X in the same buffer at RT 10 min each. The root tips were then cut just below the milky region of the tip to avoid disturbing the meristem and prepare them for size sorting and enzymatic digestion. The size-sorted root tips were then transferred into 0.05 ml transparent micro-centrifuge tubes containing enzyme solution (at least 30 µl depending on the quantity or density of the root tips in the tube). The enzyme mixture consisted of 2% cellulose RS (w/v, Yakult Pharmaceutical Ind. Co., LTD, Japan), 1% macerozyme R10 (w/v, Yakult Pharmaceutical Ind. Co., LTD, Japan), 1.5% Pectolyase Y23 (w/v, Kyowa Chemical Products, Co., LTD, Japan), 30% cellulose (v/v, C2730, Sigma, U.S.A), 30% pectinase (v/v, P2611, Sigma, U.S.A), and 40% 0.01 M Citrate buffer (pH 4.5). Enzyme incubation in a 37^o^ C water bath time varied from 20 to 30 min depending on the thickness of the root tips in the solution.

Four different pre-labeled oligonucleotides probes (POLPs) were used consisting of 18S and 5.8S rDNA 5′-labeled with AlexaFluor 488 and three different PLOPs of 5S rDNA 5′-labeled with Cy3 were combined in a probe cocktail as described elsewhere [(Islam-Faridi et al., 2020)](https://www.zotero.org/google-docs/?9UGUWx). Details of the FISH procedure used are described in [Islam-Faridi et al., 2020](https://www.zotero.org/google-docs/?1Rky7X).

The FISH slides were analyzed under a 63x plan-apochromat oil-immersion objective using an epi-fluorescence microscope (AxioImager M2, Carl Zeiss Inc., Germany) with suitable filter sets (Chroma Technology, Bellows, VT, U.S.A). The FISH images were captured with a Cool Cube 1 (MetaSystems Group Inc., Boston, MA, U.S.A) using a performance charge-coupled device (CCD) camera. Captured images were pre-processed in ISIS v5.1 (Metasystems Group Inc.) and then further processed in Adobe Photoshop CC2021 (Adobe Systems Inc., New York, NY, U.S.A) after increasing the resolution from 72 dpi (as photomicrographed) to 300 dpi.

## Comparative genomics analyses

We used OrthoFinder v2.5.2 [(Emms & Kelly, 2019)](https://www.zotero.org/google-docs/?6KWpA0) to identify gene orthogroups between *Q. rubra* and seven other plant species: *Castanea dentata* v1.1, *Castanea mollissima* [(Wang et al., 2020)](https://www.zotero.org/google-docs/?NfMvYI), *Cucumis sativus* v1.0, *Prunus persica* v2.1 [(Verde et al., 2013)](https://www.zotero.org/google-docs/?p21NKZ), *Q. robur* [(Plomion et al., 2018)](https://www.zotero.org/google-docs/?Tj0vFq), *Q. lobata* [(Sork et al., 2022)](https://www.zotero.org/google-docs/?7R9LHY), and *Q. mongolica* [(Ai et al., 2022)](https://www.zotero.org/google-docs/?y1DhN4). Primary protein sequences for *C. dentata*, *C. sativus*, *P. persica*, and *Q. rubra* were obtained from Phytozome 13 [(Goodstein et al., 2012)](https://www.zotero.org/google-docs/?7Dmarj). Primary protein sequences for *C. mollissima*, *Q. robur*, *Q. lobata*, and *Q. mongolica* were obtained from their individual sequence databases [(Ai et al., 2022; Plomion et al., 2018; Sork et al., 2022; Wang et al., 2020)](https://www.zotero.org/google-docs/?9CK4X4). The orthologous gene families and phylogenetic tree topology inferred from OrthoFinder were input to CAFE5 to identify significant expansion or contraction in each gene family (*p*<0.01) [(Mendes et al., 2020)](https://www.zotero.org/google-docs/?3DrLmg). We extracted gene names from significantly expanded and significantly contracted gene families. Then, enrichment analysis on these sets of genes was performed in Biology Network Gene Ontology Tool (BINGO) v3.0.3 [(Maere et al., 2005)](https://www.zotero.org/google-docs/?Z3Kkez) using *Q. rubra* custom annotation file as the reference provided by EnTAP v0.10.8 [(Hart et al., 2020)](https://www.zotero.org/google-docs/?xA4JcS). Hypergeometric tests along with Benjamini & Hochberg False Discovery Rate (FDR) correction (*p*<0.01) were applied in BiNGO to assess the overrepresentation of GO terms.

We used multiple methods to profile structural variation between the *Q. rubra* genome and other oak species with high-quality, chromosome-scale genomes. First, we aligned the *Q. rubra* genome to the *Q. lobata* [(Sork et al., 2022)](https://www.zotero.org/google-docs/?h5i4PG) and *Q. mongolica* [(Ai et al., 2022)](https://www.zotero.org/google-docs/?gx7igF) genomes using *nucmer* from the MUMmer4 package using *--mum -c 100* parameters [(Marçais et al., 2018)](https://www.zotero.org/google-docs/?USLMRQ). Then, the alignment block filter was applied using the *delta-filter* with one-to-one alignment mode. The nucleotide variation between the genomes was calculated using the *dnadiff* function from the MUMmer4 package. Second, we aligned the *Q. rubra* genome to the *Q. lobata* and *Q. mongolica* genomes using minimap2 v2.24. The resulting sam file was converted to a sorted bam file using Samtools v1.16.1 [(Danecek et al., 2011)](https://www.zotero.org/google-docs/?UegWYl). Then, we used Syri v1.6 to compare alignments between the genomes and to identify synteny and structural rearrangements [(Goel & Schneeberger, 2022)](https://www.zotero.org/google-docs/?2vP1bO). Lastly, Plotsr v0.5.4 was used to visualize synteny and structural rearrangements between the three oak genomes [(Goel & Schneeberger, 2022)](https://www.zotero.org/google-docs/?iXHpKb).

## Gene family comparisons

To identify and compare terpene synthase genes (TPS; PFAM: PF03936 and PF01397) from *Q. rubra* and eight other species, we first downloaded TPS amino acid sequences of *A.* *thaliana*, *E. grandis*, *P. trichocarpa*, and *V. vinifera* from [Kulheim et al., 2015](https://www.zotero.org/google-docs/?VmC0LG). For all other species (*Q. rubra, Q. robur, C. papaya*, *P. persica*, and *T. cacao*), we first manually searched their respective genome databases for keywords including ‘terpene’ and ‘terpene synthase’ to find annotated gene models. Thereafter, a BLAST search (tblastn) with one known member of each Angiosperm TPS subfamily (Type I: *TPS-c* AT4G02780, *TPS-e* AT1G79460, *TPS-f* AT1G61120, Type III: *TPS-a* AT3G32030, *TPS-b* AT3G25820, *TPS-b2* EgranTPS084, *TPS-g* EgranTPS105) was conducted to find non-annotated TPS genes and gene fragments (pseudogenes) using a cutoff Expect (E) threshold of 1e-10. Genomic coordinates from the keyword and BLAST searches were compared and redundancies were removed. Amino acid sequences of each putative TPS gene were downloaded, and in the cases where there was no gene model, the nucleotide sequence was downloaded, and 6-way translated using Expasy (<https://web.expasy.org/translate/>). Translated open reading frames were then copied to the TPS amino acid collection. To identify putatively functional TPS genes, we checked for the presence of the RLLR, DDXXD, and NSE/DTE motifs, as well as looked for evidence of gene expression where available. TPS genes missing any of these motifs, as well as showing no sign of gene expression, were marked as putative pseudogenes and excluded from further analyses. Putative functional genes smaller than 100 amino acids were also removed. This included two *V. vinifera* genes incorporated in the analyses by [Kulheim et al., 2015](https://www.zotero.org/google-docs/?2SnGNZ). Amino acid sequences from 464 putative functional TPS genes were aligned using Geneious Prime v2021.2 using muscle multiple alignment with 10,000 iterations [(Aj, 2011; Madeira et al., 2019)](https://www.zotero.org/google-docs/?wmCGsO). The alignments were manually adjusted with a focus on the three known conserved regions. The alignment was truncated to ensure that the sites were homologous. Four further *V. vinifera* sequences and one *T. cacao* sequence were removed due to insufficient sequence overlap. To create phylogeny, we first tested which amino acid substitution model had the best Akaike’s information criterion (AIC) value using Phyml’s Smart Model Selection [(Lefort et al., 2017)](https://www.zotero.org/google-docs/?xoL7TG). The model with the best AIC value was Jones-Taylor-Thornton (JTT) with gamma distribution estimated and proportion of invariable sites fixed. The phylogeny of the TPS genes was determined using 100 bootstrap replicates with the best substitution model. The phylogeny was visualized in FigTree v1.4.4 (<http://tree.bio.ed.ac.uk/software/figtree/>). TPS subfamily numbers were counted from the phylogenetic tree for each species. A gene duplication hotspot on chr 9 in *Q. rubra* between 22.65 and 24.65 Mb was visualized by depicting gene models and TPS pseudogenes across this 2 Mb stretch. From the phylogenetic tree, recent gene duplications were estimated.

IGT plant architecture genes were retrieved from the same eight assembled and annotated genomes available at Phytozome 13 [(Goodstein et al., 2012)](https://www.zotero.org/google-docs/?XUb5Sj) using *P. persica* IGT proteins as a query [(Waite & Dardick, 2021)](https://www.zotero.org/google-docs/?x3lYSM). Multiple sequence alignments were performed with the Clustal Omega web-based tools at default parameters [(Madeira et al., 2019)](https://www.zotero.org/google-docs/?TG36ds)) and visualized in Jalview v2.11.1.4. [(Procter et al., 2021)](https://www.zotero.org/google-docs/?Rq0vuG). Protein sequences were inspected for the presence of five conserved domains I-V based on signature motifs in [Waite & Dardick, 2021](https://www.zotero.org/google-docs/?7GRUjm). The maximum-likelihood phylogenetic tree was reconstructed with 100 bootstrap repeats using MEGA11 [(Tamura et al., 2021)](https://www.zotero.org/google-docs/?RoaT3b).

Using the Disease Resistance Analysis and Gene Orthology (DRAGO2) pipeline [(Osuna-Cruz et al., 2018)](https://www.zotero.org/google-docs/?zZHZcn), protein sequences from the same eight species were used to identify plant disease resistance-related domains and gene families (R-genes). DRAGO2 detects leucine rich repeat (LRR), Kinase, nucleotide-binding site (NBS)-encoding proteins, Toll/Interleukin receptor (TIR), coiled-coil (CC), and transmembrane helix (TM) domains from 60 Hidden Markov Models (HMM) modules created using HMMER v3.3.2 package [(Mistry et al., 2013)](https://www.zotero.org/google-docs/?HZ2zrc). Based on HMM motifs, genes were further categorized into five classes of R-genes: Receptor Like Kinases (RLK), Receptor Like Proteins (RLP), Coiled-coil-NBS-LRR genes (CNL), TIR-NBS-LRR genes (TNL), and other NBS-LRR genes.

## Synteny between *P. persica* v2.1 and *Q. rubra* v2.1 genomes

QTL interval for leaf emergence covers 6.18 Mb on chr 06. A region of 6.1 Mb (LG 06: 30,660,779 – 36,754,840) is syntenic to the 2 Mb-region on the top of the Prunus linkage group 1 (Pp01:17,76,889 -37,46,581). Additionally, a small region of 180 kb (LG 06: 36,766,083 – 36,945,618) is syntenic to 106 kb pericentromeric region on the same peach pseudochromosome (Pp 01:22,208,427-22,314,659). On the top of the Prunus G1, QTLs for chill requirement and bloom date were reported in F2 cross composed of 378 individuals. Seedlings were propagated in three biological replicates and phenotyped multiple years (Fan et al., 2010; Zhebentyayeva et al., 2014). The QTLs for bloom date and growing degree hours to flowering were also reported in F2 peach x almond cross (Cantin et al., 2020).

## Population structure and local adaptation

High molecular weight genomic DNAs were prepared (as per section 1.2) and delivered to the MycroArray Company for exome-capture. Using *Q. rubra* reference transcriptome generated by [Soltani et al., 2020](https://www.zotero.org/google-docs/?qnTt1h), we selected a set of 20,000 optimal baits (80 nucleotides each), from a set of 618 K candidates provided by the MycroArray Company, for targeted exome sequence capture. Using the 20,000 selected baits, the MycroArray Company constructed exome-capture libraries from each of the 96 *Q. rubra* DNAs, which were provided as a single titrated pool of DNA libraries for sequencing. Sequencing of the exome-captured library pool was conducted by the Penn State University Genome Center on an Illumina HiSeq 2500 machine, producing 1x150 bp reads.

Exome capture datasets were processed following the pipeline by [(Harvey et al., 2016)](https://www.zotero.org/google-docs/?hqa8WU). Briefly, after QC with FastQC v0.11.7 (Andrews, 2017), clean reads (Trimmomatic v0.33; [(Bolger et al., 2014)](https://www.zotero.org/google-docs/?7kn89r)) were aligned against the *Q. rubra* genome using BWA v0.7.15 [(Li & Durbin, 2009)](https://www.zotero.org/google-docs/?dd9ixJ). Indexed and properly aligned reads (-q 20) were retained and marked for PCR duplicates with Picard v2.20.8 (http://broadinstitute.github.io/picard/) and Samtools v1.5 [(Danecek et al., 2011)](https://www.zotero.org/google-docs/?hqKBjl). Individual bam files having 4.6 million reads per file on average were merged using the *MergeSamFiles* command in Picard. SNP genotyping was conducted using the UnifiedGenotyper implemented in GenomeAnalysisTK-3.3-0 [(Poplin et al., 2017)](https://www.zotero.org/google-docs/?kNdXVp). Genotypic data files were filtered for biallelic SNPs only, missing rate across accessions less than 25%, and minor allele frequency (MAF) more than 5% using Bcftools v1.10.2 [(Danecek et al., 2011)](https://www.zotero.org/google-docs/?eewYb1), Vcftools v0.1.16 [(Danecek et al., 2011)](https://www.zotero.org/google-docs/?fMP5I6), and Vcflib v1.0.2 [(Garrison et al., 2021)](https://www.zotero.org/google-docs/?I4BIAg), consecutively. At the data processing stage, three accessions were removed from downstream analyses due to the poor quality of mapped reads. To phase parental chromosomes in 3-member maternal half-sib families, we used the R package ‘hsphase’ [(Ferdosi et al., 2014)](https://www.zotero.org/google-docs/?7uIhM5).

We checked accessions for relatedness to geographically distant accessions and removed three more such accessions that suggested inaccurate seed sources or DNA sample provenance, leaving us with 157,237 SNPs with an MAF of at least 0.05 for 90 samples. To assess population structure, we calculated the pairwise genetic distances between these samples using PLINK and then generated a multidimensional scaling of these distances in 2 dimensions [(Purcell et al., 2007)](https://www.zotero.org/google-docs/?6wqWNm).

To identify individual loci associated with local adaptation, we used two complementary approaches. First, we used the R package ‘hierfstat’ [(Goudet, 2005)](https://www.zotero.org/google-docs/?IlsKk4) to calculate *F_ST_* values between the four sampled geographic population clusters: Southern Appalachian, Indiana, Pennsylvania, and Canada. Second, we implemented redundancy analysis (RDA), a multivariate ordination approach that maximizes the proportion of SNP variation explained by linear combinations of environmental variables. We used nine environmental variables that we hypothesized to be ecologically important and had relatively low correlations, including mean diurnal temperature range, minimum temperature of the coldest month, precipitation seasonality, the mean temperature of the warmest quarter, and elevation from CHELSA [(Brun et al., 2022)](https://www.zotero.org/google-docs/?3m4PBF) as well as annual aridity index and the aridity index of the driest month [(Zomer et al., 2008)](https://www.zotero.org/google-docs/?87COFb), soil moisture capacity [(Dunne & Willmott, 2000)](https://www.zotero.org/google-docs/?VclmE9) and depth to water table [(Fan et al., 2013)](https://www.zotero.org/google-docs/?IN86Lz). To impute missing SNP calls for RDA, we used the average allele frequency at each locus. We then identified loci that loaded strongest on RDA canonical axes, i.e., where allele frequency was strongly associated with the linear combination of environmental variables loading onto these axes. To validate whether the environmental ordination of the RDA captured an important component of local adaptation, we calculated the distance between the provenance trial (Vallonia, IN, U.S.A) and the site of origin for each tree, along the first two axes of linear combinations of environment.

We phenotyped trees in July 2016 at the sampled common garden in Vallonia, IN. We measured height and diameter at breast height (DBH) to assess performance. We also measured traits on leaves. We scanned leaves with a LI-3100C leaf area meter (LI-COR). We oven dried and weighed leaves to then calculate specific leaf area (SLA). We used ~3-4 mg of dried leaf material per tree and sent it to the UC-Davis Stable Isotope Facility for mass spectrometry measurement of total C, total N, δ^13^C, and δ^15^N estimation. We also took nail polish impressions of 2 locations on each of 2 leaves from each tree and counted stomata under a stereoscope in a 0.0625 mm^2^ box. 

## Estimation of nucleotide diversity

After trimming and removing low-quality reads, 175 million of 150 bp paired-end reads were retained. The quality of reads was assessed with Fastqc v0.11.4 [(Andrews, 2017)](https://www.zotero.org/google-docs/?pUOKkW). Raw reads were mapped against the *Q. rubra* genome using BWA-MEM v0.7.12 [(Li & Durbin, 2009)](https://www.zotero.org/google-docs/?5sjytC). Sorting of reads and removal of PCR duplicates were performed with Samtools v1.9 [(Danecek et al., 2021)](https://www.zotero.org/google-docs/?AKvInI). Variant sites were called using Bcftools mpileup v1.9 [(Danecek et al., 2021)](https://www.zotero.org/google-docs/?tFC9RU). After reviewing descriptive statistics, the data filtering steps were conducted using Vcftools v0.1.16 [(Danecek et al., 2011)](https://www.zotero.org/google-docs/?4R90D7). We removed all variants with depth lower than 10 and higher than 50 and quality lower than 30. All variants with more than 10% of missing data and all indels were removed. The final data set for nucleotide diversity calculation consisted of 51.8 million positions including monomorphic sites. For *F_ST_* outlier detection analysis, only bi-allelic sites were kept and a Minor Allele Frequency (MAF) filter of 0.05 was applied to generate the data set of 5.9 million SNPs. Genes from the *Q. rubra* genome annotation were used for visualizing gene density. From the same annotation file, parts of the genome were extracted to calculate nucleotide diversity in coding versus non-coding genomic regions. For calculating *F_ST_* and nucleotide diversity, Vcftools v0.1.16 was used [(Danecek et al., 2011)](https://www.zotero.org/google-docs/?6xwKLa). Nucleotide diversity was calculated over a window size of 10 Kb while *F_ST_* was calculated on a SNP-by-SNP basis. The significance of differences between the two populations was checked using Wilcoxon ranked-sum tests. Visualization of results was done in R v3.6.1 [(R Core Team, 2013)](https://www.zotero.org/google-docs/?0uk7uR).

# Supplementary Results

## Candidate genes within leaf emergence QTL interval

### Plant hormones (8 genes)

In total, 46 genes within QTL are potential candidates for leaf emergence in *Q. rubra* genome. Of these, five genes (*Qurub.06G151000*, *Qurub.06G153800*, *Qurub.06G159200*, *Qurub.06G162600*, *Qurub.06G166200*) were annotated as MYB-like DNA-binding proteins - some of them are homologs of transcriptional activators induced by ABA or regulators of cold tolerance in *A. thaliana.* Group of four ethylene responsive factors includes the ERF1 homologs (*Qurub.06G165700*, *Qurub.06G165800*, *Qurub.06G165900*) - some of them delay flowering through direct inhibition of FLOWERING LOCUS T expression in Arabidopsis (Chen et al., 2021). Putative ethylene response sensor related protein (*Qurub.06G169400*) represents membrane-bound histidine kinase mediates the cellular response, mostly through differential expression of target genes (Hua et al., 1998). The *Qurub.06G166400* was annotated as putative homolog of a basic helix-loop-helix 104 (bHLH104) protein that is a transcriptional repressor for glucose and abscisic acid signaling pathways in Arabidopsis (Min et al., 2019).

### Chromatin remodelling (9 genes)

Several genes within QTL interval imply epigenetic control of plant development via chromatin modifications. Two putative histone methyltransferases *Qurub.06G151600* and *Qurub.06G155100*, homologs of the SET domain proteins SUVH9 (AT4G13460) and SUVR4 (AT3G0438) from *A. thaliana,* play a central role in histone-mediated gene silencing via the H3K9m methylation and heterochromatin formation (Fisher et al., 2009; El-Sappah et al., 2021). Putative protein *Qurub.06G162700*, a subunit of the NUA4 histone acetyltransferase complex, which involved in transcriptional gene activation by acetylation of nucleosomal histones H4 and H2A (Altaf et al., 2010; Bieluszewski et al., 2022). Two tandem duplicated putative Nuclear Actin-Related Proteins *Qurub.06G168200* and *Qurub.06G168600* are orthologs of the actin-related protein 9 (AT5G43500, ARP9) from *A. thaliana*, one of subunit of a protein complex of the Ino80p ATPase with chromatin remodeling activity (Shang et al., 2021; Yang et al., 2022).

Putative SNF5-type protein *Qurub.06G176400*, an ortholog of the BUSHY (AT3G17590), is conserved subunit of the ATP-dependent nucleosome-remodelling complex SWI/SNF. In Arabidopsis, it regulates a seed germination via the gibberellin pathway and responds to environmental changes in temperature-dependent manner (Gratkowska-Zmuda et al., 2020; Wang et al., 2022). Putative C3HC4 RING-domain-containing ubiquitin E3 ligase *Qurub.06G173600* is a component of the PRC1-like polycomb repressive complex DREB2H, a key transcriptional activator responding to drought and heat (Qin et al., 2008; rev. Merini and Calonje, 2015). Putative helicase protein with RING/U-box domain Qurub.06G168800 was annotated a homolog of the class SWItch/Sucrose Non-Fermentable (SWI/SNF) ATPase AtRAD5B (AT5G43530). This enzyme is involved in a G-protein signaling pathway which regulates auxin gradient and transport. Auxin gradients are known to recruit SWI/SNF remodeling complexes to the chromatin and regulate expression of genes involved in flower and leaf formation (Khatri et al. 2017). Predicted regulator of chromosome condensation family protein RCC1 (*Qurub.06G161900*) belong to gene family that plays dynamic but unknown role in maintaining chromosome and nuclear structure (Dasso, 1993).

### Transcriptional factors – stimuli responsive (3 genes)

Putative transducin/WD40 repeat-like superfamily protein *Qurub.06G158800*, homolog of protein encoded by AT4G14310 from Arabidopsis, which has a role in cell cycle phase transition and cell division (Van Leene et al., 2010). Predicted protein *Qurub.06G172700* is putative ortholog of the basic helix-loop-helix transcriptional factor 92 (bHLH92) from *A. thaliana* (AT5G43650). In sheepgrass, the LcbHLH92 acts as a negative regulator of anthocyanin/proanthocyandin accumulation and influences seed dormancy (Zhao et al., 2019).

### Light sensing -Photoperiod receptor- circadian clocks

Predicted gene *Qurub.06G176100* encodes a homolog of ELONGATED HYPOCOTYL 5 (HY5), a bZIP-type transcription factor involved in a phyB signaling pathway (Kurihara et al., 2020; Marzi et al, 2020). Peach HY5 orthologs, PpHY5 and PpHYH, also regulate anthocyanin accumulation in response to UV irradiation (Zhao et al., 2021). In tomato, HY5 ortholog regulates seedling photomorphogenesis and accumulation phenylpropanoid metabolites in fruits (Zhang et al. 2022).

Putative protein *Qurub.06G162900* is a homolog of the photoperiod receptor CRYPTOCHROME2 (ATCRY2), which mediates a blue-light dependent cotyledon expansion and flowering time (Olsen et al., 2004). In Arabidopsis, it is a positive regulator of the central component of CIRCADIAN CLOCK ASSOCIATED 1 (CCA1) gene encoding a central component of the circadian oscillator (Mo et al., 2022). In tomato CR1and CRY2 proteins play a pivotal role in plant development including flowering time (Fantini et al. 2019). In total three cryptochromes (CRYs) were predicted in the *Q. rubra* genome - *Qurub.12G173700* on chr12 (LG12: 32,881,215-32,890,441 forward) and *Qurub.05G185000* (LG5 : 44,707,877-44,721,075 reverse). Similarly, the *A. thaliana* genome contains three CRY genes, two CRYs (CRY1 and CRY2) of the plant CRY subfamily, and one CRY (CRY3) of the CRY-DASH subfamily. The CRY1 and CRY2 possess partially redundant and overlapping functions (Mo et al., 2022).

CRYs mediate H2A.Z deposition at the HY5 target genes promoting cell elongation (Mao et al. 2021). Response to light of purified Arabidopsis Cry1 and Cry2 proteins was significantly altered by temperature (Pooam et al. 2021). Light Quality Modulates Plant Cold Response and Freezing (Kameniarová et al., 2022).

# Supplemental Figures

**
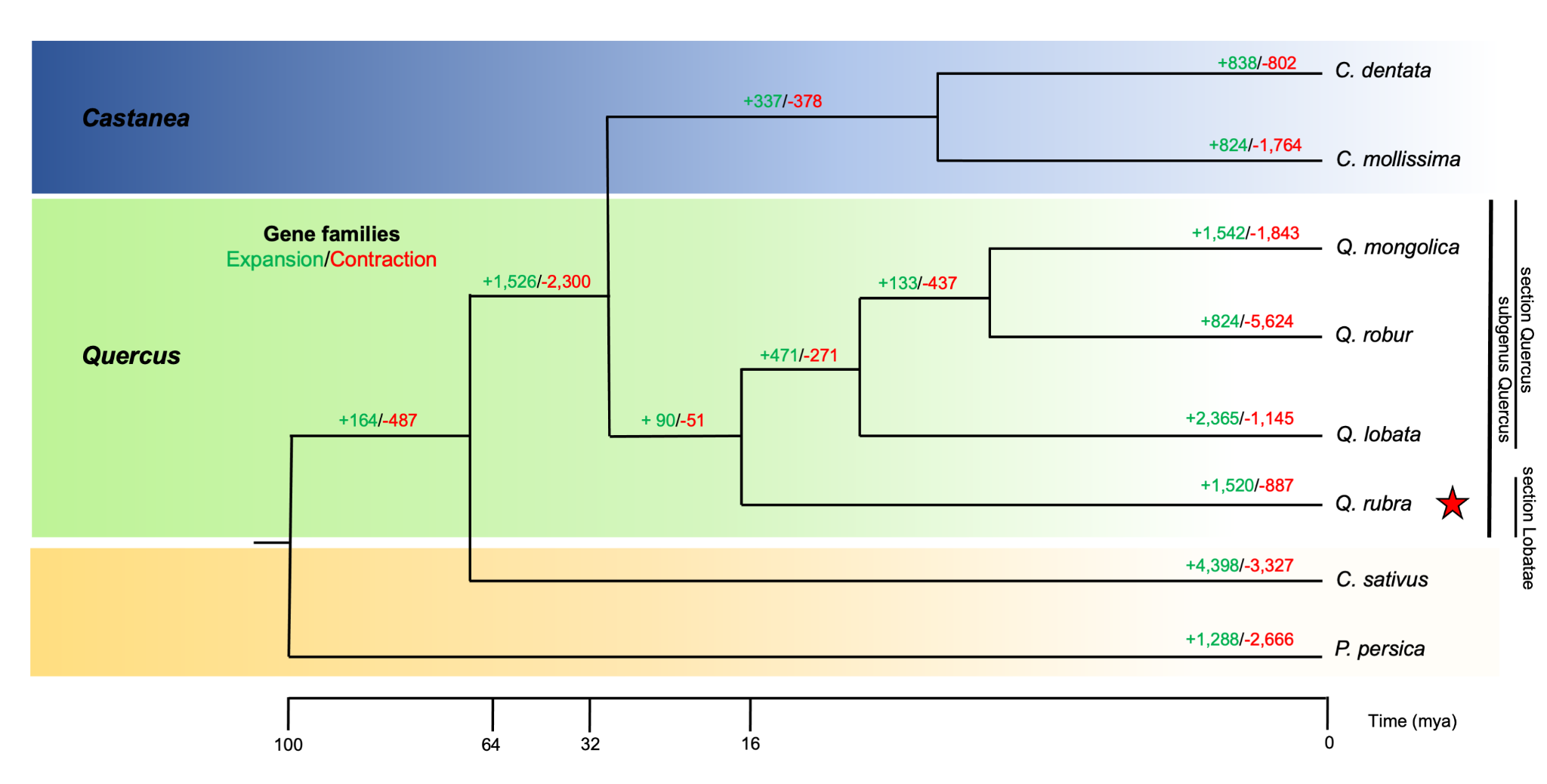
**

**Figure S1.** Evolutionary history and expanded and contracted gene families in *Q. rubra* (red star) and seven plant species (*C. dentata*, *C. mollissima*, *Q. mongolica*, *Q. robur*, *Q. lobata*, *C. sativus*, and *P. persica*). Blue color block represents *Castanea* spp., green color block represents *Quercus* spp., and yellow block contains two outgroups, *C. sativus*, and *P. persica*.


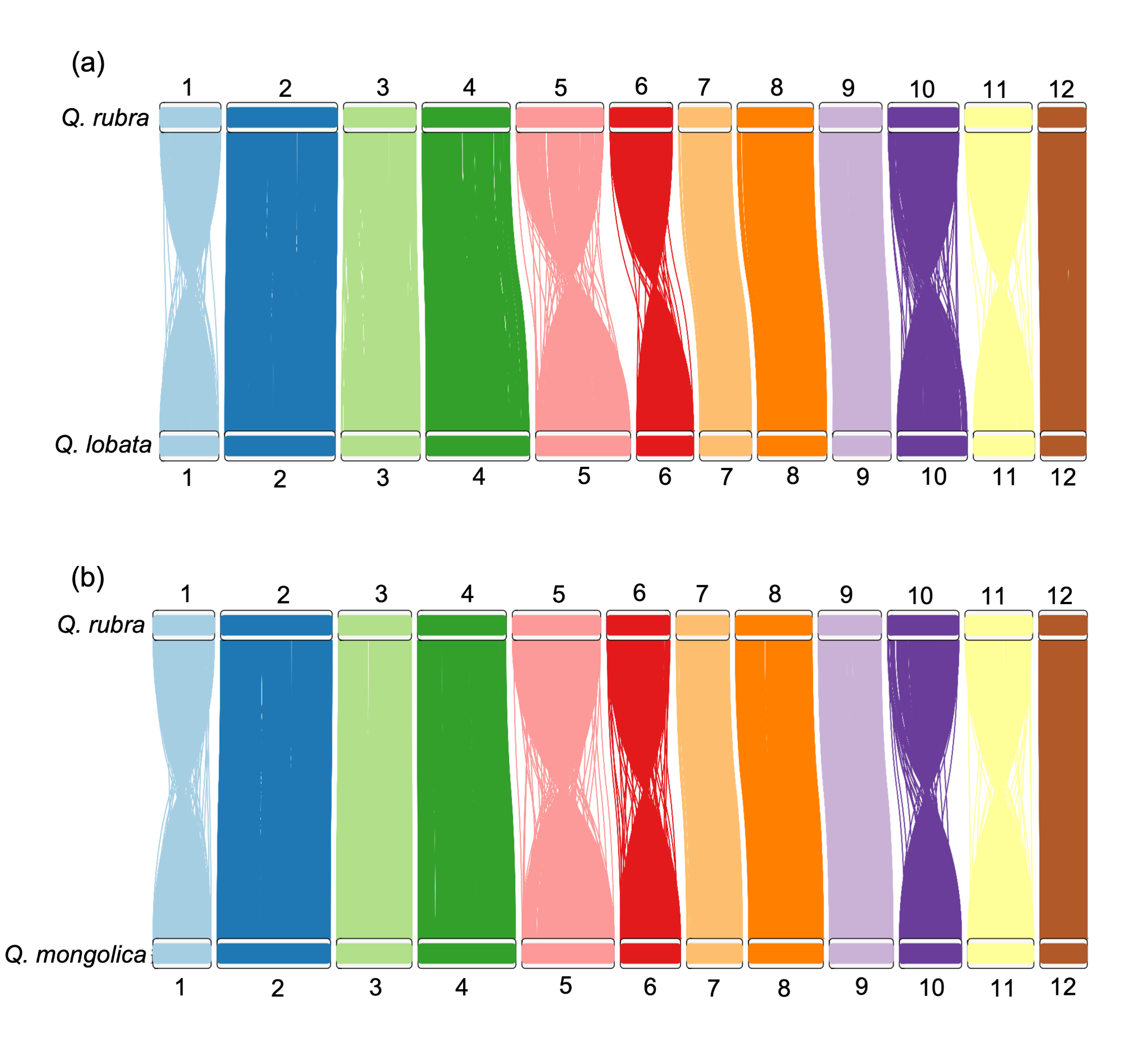


**Figure S2.** Alignment of protein sequences of (a) *Q. rubra* and *Q. lobata* genomes and (b) *Q. rubra* and *Q. mongolica* genomes. *Chromosomes 1, 5, 6, 10, and 11 are reversed in *Q. rubra* genome, relative to *Q. lobata* and *Q. mongolica*, to be consistent with the previously published *Q. rubra* genetic map (Konar et al., 2017).

**
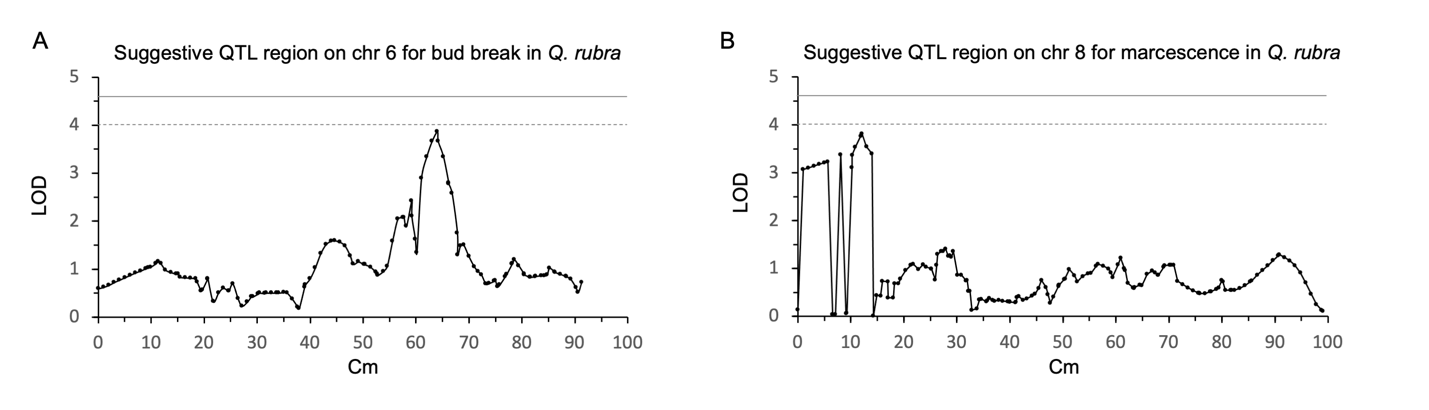
**

**Figure S3.** (a) Distribution of LOD scores on chr 6 for bud break. LOD scores on the other 11 linkage groups were ≤ 2.61. (b) Distribution of LOD scores on chr 8 for marcescence. LOD scores on the other 11 linkage groups were ≤ 3.1. The dotted indicates a P-value of 0.15, the solid line a P-value of 0. 05.

**
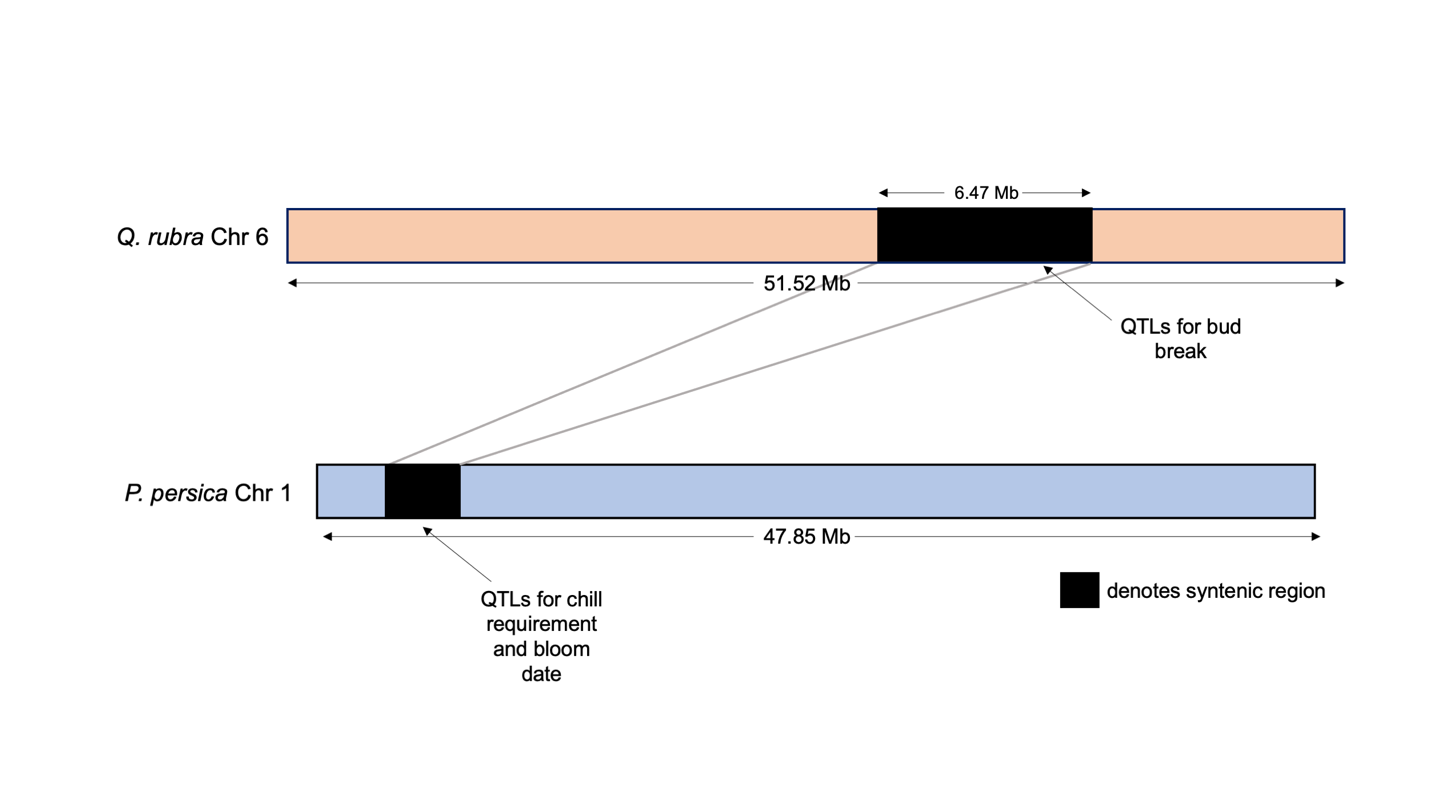
**

**Figure S4.** Synteny of bud break QTL region in *Q. rubra* chr 6 to one end of the *P. persica* chr 1 with QTLs for chill requirement and bloom date.

**
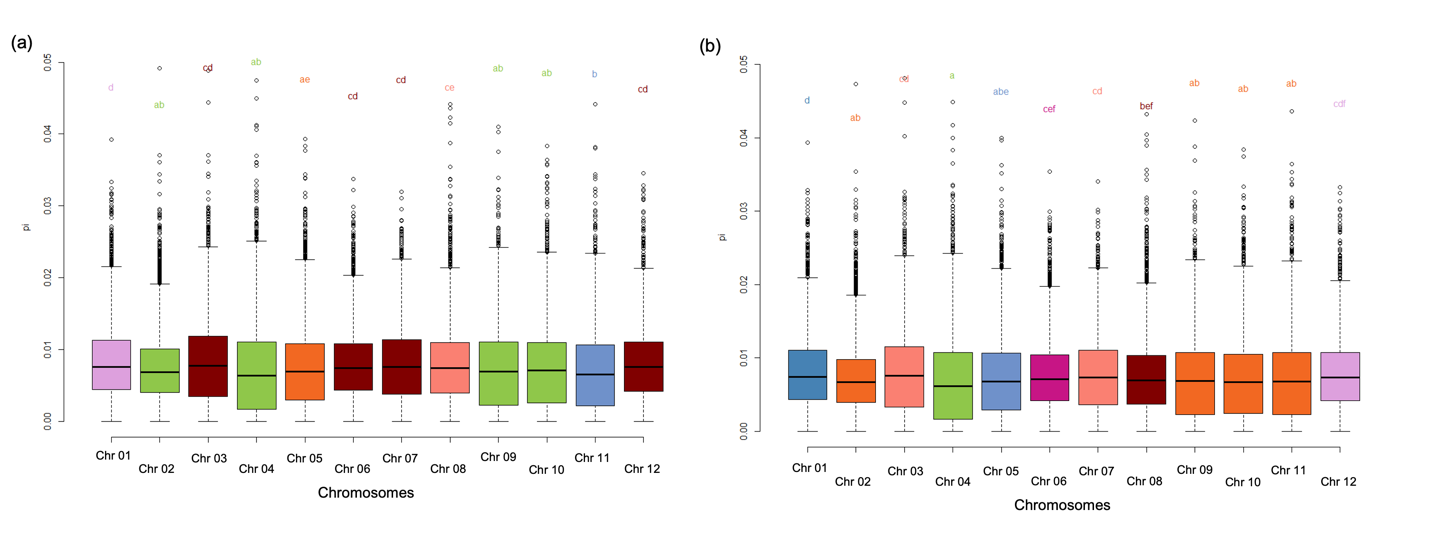
Figure S5.** Variation of nucleotide genetic diversity across chromosomes at the population level. A. Population Lisle. B. Population Covington. Colors correspond to Tukey's Honestly Significant Difference criterion at the α = 0.05 significance level. The chromosomes with the same letters and colors are not statistically different in nucleotide diversity. The bottom and top of the box are the 25th and 75th percentile. The band near the middle of the box is the 50th percentile. For the ends of the whiskers, the default box plot parameter for statistical dispersion in R was used.

**
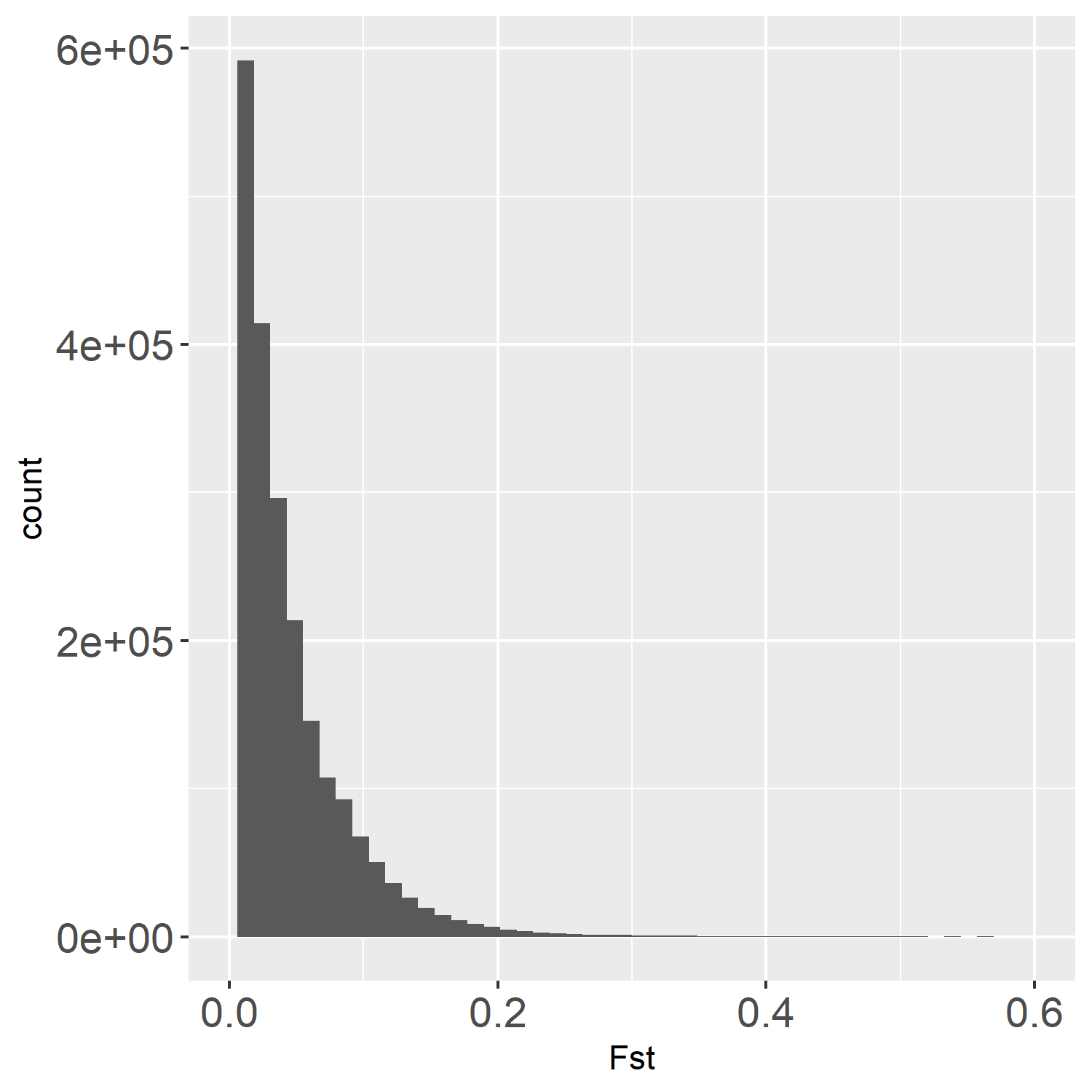
**

**Figure S6.** Empirical distribution of the *F_ST_* values across the SNPs between Lisle and Covington populations.

1. **References**

[Ai, W., Liu, Y., Mei, M., Zhang, X., Tan, E., Liu, H., Han, X., Zhan, H., & Lu, X. (2022). A chromosome-scale genome assembly of the mongolian oak (*Quercus mongolica*). *Molecular Ecology Resources*, *22*(6), 2396–2410. https://doi.org/10.1111/1755-0998.13616](https://www.zotero.org/google-docs/?ShzSJ8)

[Aj, D. (2011). Geneious v5.4. *Http://Www.Geneious.Com*. https://cir.nii.ac.jp/crid/1573387449123163648](https://www.zotero.org/google-docs/?ShzSJ8)

[Altschul, S. F., Gish, W., Miller, W., Myers, E. W., & Lipman, D. J. (1990). Basic local alignment search tool. *Journal of Molecular Biology*, *215*(3), 403–410. https://doi.org/10.1016/S0022-2836(05)80360-2](https://www.zotero.org/google-docs/?ShzSJ8)

[Andrews, S. (2017). FastQC: a quality control tool for high throughput sequence data.](https://www.zotero.org/google-docs/?ShzSJ8)

[Boeckmann, B., Bairoch, A., Apweiler, R., Blatter, M.-C., Estreicher, A., Gasteiger, E., Martin, M. J., Michoud, K., O’Donovan, C., & Phan, I. (2003). The SWISS-PROT protein knowledgebase and its supplement TrEMBL in 2003. *Nucleic Acids Research*, *31*(1), 365–370.](https://www.zotero.org/google-docs/?ShzSJ8)

[Bolger, A. M., Lohse, M., & Usadel, B. (2014). Trimmomatic: A flexible trimmer for Illumina sequence data. *Bioinformatics*, *30*(15), 2114–2120.](https://www.zotero.org/google-docs/?ShzSJ8)

[Brun, P., Zimmermann, N. E., Hari, C., Pellissier, L., & Karger, D. N. (2022). Global climate-related predictors at kilometer resolution for the past and future. *Earth System Science Data*, *14*(12), 5573–5603. https://doi.org/10.5194/essd-14-5573-2022](https://www.zotero.org/google-docs/?ShzSJ8)

[Callahan, A. M., Zhebentyayeva, T. N., Humann, J. L., Saski, C. A., Galimba, K. D., Georgi, L. L., Scorza, R., Main, D., & Dardick, C. D. (2021). Defining the ‘HoneySweet’ insertion event utilizing NextGen sequencing and a de novo genome assembly of plum (*Prunus domestica*). *Horticulture Research*, *8*, 8. https://doi.org/10.1038/s41438-020-00438-2](https://www.zotero.org/google-docs/?ShzSJ8)

[Coggeshall, M. (1993). Oak tree improvement in Indiana. *Annales Des Sciences Forestières*, *50*(Suppl1), 416s–419s.](https://www.zotero.org/google-docs/?ShzSJ8)

[Danecek, P., Auton, A., Abecasis, G., Albers, C. A., Banks, E., DePristo, M. A., Handsaker, R. E., Lunter, G., Marth, G. T., Sherry, S. T., McVean, G., Durbin, R., & 1000 Genomes Project Analysis Group. (2011). The variant call format and VCFtools. *Bioinformatics*, *27*(15), 2156–2158. https://doi.org/10.1093/bioinformatics/btr330](https://www.zotero.org/google-docs/?ShzSJ8)

[Danecek, P., Bonfield, J. K., Liddle, J., Marshall, J., Ohan, V., Pollard, M. O., Whitwham, A., Keane, T., McCarthy, S. A., Davies, R. M., & Li, H. (2021). Twelve years of SAMtools and BCFtools. *GigaScience*, *10*(2), giab008. https://doi.org/10.1093/gigascience/giab008](https://www.zotero.org/google-docs/?ShzSJ8)

[Dunne, K. A., & Willmott, C. J. (2000). Global Distribution of Plant-Extractable Water Capacity of Soil (Dunne). *ORNL DAAC*. https://doi.org/10.3334/ORNLDAAC/545](https://www.zotero.org/google-docs/?ShzSJ8)

[Emms, D. M., & Kelly, S. (2019). OrthoFinder: Phylogenetic orthology inference for comparative genomics. *Genome Biology*, *20*(1), 1–14.](https://www.zotero.org/google-docs/?ShzSJ8)

[Fan, Y., Li, H., & Miguez-Macho, G. (2013). Global patterns of groundwater table depth. *Science*, *339*(6122), 940–943. https://doi.org/10.1126/science.1229881](https://www.zotero.org/google-docs/?ShzSJ8)

[Ferdosi, M. H., Kinghorn, B. P., van der Werf, J. H., Lee, S. H., & Gondro, C. (2014). hsphase: An R package for pedigree reconstruction, detection of recombination events, phasing and imputation of half-sib family groups. *BMC Bioinformatics*, *15*(1), 172. https://doi.org/10.1186/1471-2105-15-172](https://www.zotero.org/google-docs/?ShzSJ8)

[Flynn, J. M., Hubley, R., Goubert, C., Rosen, J., Clark, A. G., Feschotte, C., & Smit, A. F. (2020). RepeatModeler2 for automated genomic discovery of transposable element families. *Proceedings of the National Academy of Sciences*, *117*(17), 9451–9457. https://doi.org/10.1073/pnas.1921046117](https://www.zotero.org/google-docs/?ShzSJ8)

[Forester, B. R., Lasky, J. R., Wagner, H. H., & Urban, D. L. (2018). Comparing methods for detecting multilocus adaptation with multivariate genotype–environment associations. *Molecular Ecology*, *27*(9), 2215–2233. https://doi.org/10.1111/mec.14584](https://www.zotero.org/google-docs/?ShzSJ8)

[Garrison, E., Kronenberg, Z. N., Dawson, E. T., Pedersen, B. S., & Prins, P. (2021). Vcflib and tools for processing the VCF variant call format. *BioRxiv*.](https://www.zotero.org/google-docs/?ShzSJ8)

[Goel, M., & Schneeberger, K. (2022). plotsr: Visualizing structural similarities and rearrangements between multiple genomes. *Bioinformatics*, *38*(10), 2922–2926. https://doi.org/10.1093/bioinformatics/btac196](https://www.zotero.org/google-docs/?ShzSJ8)

[Goodstein, D. M., Shu, S., Howson, R., Neupane, R., Hayes, R. D., Fazo, J., Mitros, T., Dirks, W., Hellsten, U., Putnam, N., & Rokhsar, D. S. (2012). Phytozome: A comparative platform for green plant genomics. *Nucleic Acids Research*, *40*(D1), D1178–D1186. https://doi.org/10.1093/nar/gkr944](https://www.zotero.org/google-docs/?ShzSJ8)

[Goudet, J. (2005). Hierfstat, a package for r to compute and test hierarchical F-statistics. *Molecular Ecology Notes*, *5*(1), 184–186. https://doi.org/10.1111/j.1471-8286.2004.00828.x](https://www.zotero.org/google-docs/?ShzSJ8)

[Haas, B. J., Delcher, A. L., Mount, S. M., Wortman, J. R., Smith, R. K., Hannick, L. I., Maiti, R., Ronning, C. M., Rusch, D. B., Town, C. D., Salzberg, S. L., & White, O. (2003). Improving the Arabidopsis genome annotation using maximal transcript alignment assemblies. *Nucleic Acids Research*, *31*(19), 5654–5666. https://doi.org/10.1093/nar/gkg770](https://www.zotero.org/google-docs/?ShzSJ8)

[Hart, A. J., Ginzburg, S., Xu, M. (Sam), Fisher, C. R., Rahmatpour, N., Mitton, J. B., Paul, R., & Wegrzyn, J. L. (2020). EnTAP: Bringing faster and smarter functional annotation to non-model eukaryotic transcriptomes. *Molecular Ecology Resources*, *20*(2), 591–604. https://doi.org/10.1111/1755-0998.13106](https://www.zotero.org/google-docs/?ShzSJ8)

[Harvey, M. G., Smith, B. T., Glenn, T. C., Faircloth, B. C., & Brumfield, R. T. (2016). Sequence capture versus restriction site associated DNA sequencing for shallow systematics. *Systematic Biology*, *65*(5), 910–924.](https://www.zotero.org/google-docs/?ShzSJ8)

[Islam-Faridi, N., Mason, M. E., Koch, J. L., & Nelson, C. D. (2020). Cytogenetics of Fraxinus mandshurica and F. quadrangulata: Ploidy determination and rDNA analysis. *Tree Genetics & Genomes*, *16*(1), 1–7. https://doi.org/10.1007/s11295-020-1418-6](https://www.zotero.org/google-docs/?ShzSJ8)

[Jewell, D. C., & Islam-Faridi, N. (1994). A Technique for Somatic Chromosome Preparation and C-banding of Maize. In M. Freeling & V. Walbot (Eds.), *The Maize Handbook* (pp. 484–493). Springer. https://doi.org/10.1007/978-1-4612-2694-9_75](https://www.zotero.org/google-docs/?ShzSJ8)

[Konar, A., Choudhury, O., Bullis, R., Fiedler, L., Kruser, J. M., Stephens, M. T., Gailing, O., Schlarbaum, S., Coggeshall, M. V., Staton, M. E., Carlson, J. E., Emrich, S., & Romero-Severson, J. (2017). High-quality genetic mapping with ddRADseq in the non-model tree Quercus rubra. *BMC Genomics*, *18*(1), 417. https://doi.org/10.1186/s12864-017-3765-8](https://www.zotero.org/google-docs/?ShzSJ8)

[Krzywinski, M., Schein, J., Birol, İ., Connors, J., Gascoyne, R., Horsman, D., Jones, S. J., & Marra, M. A. (2009). Circos: An information aesthetic for comparative genomics. *Genome Research*, *19*(9), 1639–1645. https://doi.org/10.1101/gr.092759.109](https://www.zotero.org/google-docs/?ShzSJ8)

[Kubisiak, T. L., Nelson, C. D., Staton, M. E., Zhebentyayeva, T., Smith, C., Olukolu, B. A., Fang, G.-C., Hebard, F. V., Anagnostakis, S., Wheeler, N., Sisco, P. H., Abbott, A. G., & Sederoff, R. R. (2013). A transcriptome-based genetic map of Chinese chestnut (*Castanea mollissima*) and identification of regions of segmental homology with peach (*Prunus persica*). *Tree Genetics & Genomes*, *9*(2), 557–571. https://doi.org/10.1007/s11295-012-0579-3](https://www.zotero.org/google-docs/?ShzSJ8)

[Kulheim, C., Padovan, A., Hefer, C., Krause, S. T., Köllner, T. G., Myburg, A. A., Degenhardt, J., & Foley, W. J. (2015). The Eucalyptus terpene synthase gene family. *BMC Genomics*, *16*(1), 450. https://doi.org/10.1186/s12864-015-1598-x](https://www.zotero.org/google-docs/?ShzSJ8)

[Lagesen, K., Hallin, P., Rødland, E. A., Staerfeldt, H.-H., Rognes, T., & Ussery, D. W. (2007). RNAmmer: Consistent and rapid annotation of ribosomal RNA genes. *Nucleic Acids Research*, *35*(9), 3100–3108. https://doi.org/10.1093/nar/gkm160](https://www.zotero.org/google-docs/?ShzSJ8)

[Lefort, V., Longueville, J.-E., & Gascuel, O. (2017). SMS: Smart Model Selection in PhyML. *Molecular Biology and Evolution*, *34*(9), 2422–2424. https://doi.org/10.1093/molbev/msx149](https://www.zotero.org/google-docs/?ShzSJ8)

[Li, H. (2018). Minimap2: Pairwise alignment for nucleotide sequences. *Bioinformatics*, *34*(18), 3094–3100. https://doi.org/10.1093/bioinformatics/bty191](https://www.zotero.org/google-docs/?ShzSJ8)

[Li, H., & Durbin, R. (2009). Fast and accurate short read alignment with Burrows–Wheeler transform. *Bioinformatics*, *25*(14), 1754–1760.](https://www.zotero.org/google-docs/?ShzSJ8)

[Ma, X., Olsen, J. L., Reusch, T. B. H., Procaccini, G., Kudrna, D., Williams, M., Grimwood, J., Rajasekar, S., Jenkins, J., Schmutz, J., & Peer, Y. V. de. (2021). *Improved chromosome-level genome assembly and annotation of the seagrass,* Zostera marina *(eelgrass)* (10:289). F1000Research. https://doi.org/10.12688/f1000research.38156.1](https://www.zotero.org/google-docs/?ShzSJ8)

[Madeira, F., Park, Y. mi, Lee, J., Buso, N., Gur, T., Madhusoodanan, N., Basutkar, P., Tivey, A. R. N., Potter, S. C., Finn, R. D., & Lopez, R. (2019). The EMBL-EBI search and sequence analysis tools APIs in 2019. *Nucleic Acids Research*, *47*(W1), W636–W641. https://doi.org/10.1093/nar/gkz268](https://www.zotero.org/google-docs/?ShzSJ8)

[Maere, S., Heymans, K., & Kuiper, M. (2005). BiNGO: A Cytoscape plugin to assess overrepresentation of Gene Ontology categories in Biological Networks. *Bioinformatics*, *21*(16), 3448–3449. https://doi.org/10.1093/bioinformatics/bti551](https://www.zotero.org/google-docs/?ShzSJ8)

[Marçais, G., Delcher, A. L., Phillippy, A. M., Coston, R., Salzberg, S. L., & Zimin, A. (2018). MUMmer4: A fast and versatile genome alignment system. *PLOS Computational Biology*, *14*(1), e1005944. https://doi.org/10.1371/journal.pcbi.1005944](https://www.zotero.org/google-docs/?ShzSJ8)

[Martin, M., Patterson, M., Garg, S., Fischer, S., Pisanti, N., Klau, G. W., Schöenhuth, A., & Marschall, T. (2016). WhatsHap: Fast and accurate read-based phasing. *BioRxiv*, 085050.](https://www.zotero.org/google-docs/?ShzSJ8)

[Mendes, F. K., Vanderpool, D., Fulton, B., & Hahn, M. W. (2020). CAFE 5 models variation in evolutionary rates among gene families. *Bioinformatics*.](https://www.zotero.org/google-docs/?ShzSJ8)

[Mistry, J., Finn, R. D., Eddy, S. R., Bateman, A., & Punta, M. (2013). Challenges in homology search: HMMER3 and convergent evolution of coiled-coil regions. *Nucleic Acids Research*, *41*(12), e121. https://doi.org/10.1093/nar/gkt263](https://www.zotero.org/google-docs/?ShzSJ8)

[Osuna-Cruz, C. M., Paytuvi-Gallart, A., Di Donato, A., Sundesha, V., Andolfo, G., Aiese Cigliano, R., Sanseverino, W., & Ercolano, M. R. (2018). PRGdb 3.0: A comprehensive platform for prediction and analysis of plant disease resistance genes. *Nucleic Acids Research*, *46*(D1), D1197–D1201. https://doi.org/10.1093/nar/gkx1119](https://www.zotero.org/google-docs/?ShzSJ8)

[Ou, S., Chen, J., & Jiang, N. (2018). Assessing genome assembly quality using the LTR Assembly Index (LAI). *Nucleic Acids Research*, *46*(21), e126–e126.](https://www.zotero.org/google-docs/?ShzSJ8)

[Pacific Biosciences. (2014). Guidelines for using a salt: Chloroform wash to clean up gDNA. Pacific Biosciences of California.](https://www.zotero.org/google-docs/?ShzSJ8)

[Plomion, C., Aury, J.-M., Amselem, J., Leroy, T., Murat, F., Duplessis, S., Faye, S., Francillonne, N., Labadie, K., Le Provost, G., Lesur, I., Bartholomé, J., Faivre-Rampant, P., Kohler, A., Leplé, J.-C., Chantret, N., Chen, J., Diévart, A., Alaeitabar, T., … Salse, J. (2018). Oak genome reveals facets of long lifespan. *Nature Plants*, *4*(7), 440–452. https://doi.org/10.1038/s41477-018-0172-3](https://www.zotero.org/google-docs/?ShzSJ8)

[Poplin, R., Ruano-Rubio, V., DePristo, M. A., Fennell, T. J., Carneiro, M. O., Van der Auwera, G. A., Kling, D. E., Gauthier, L. D., Levy-Moonshine, A., & Roazen, D. (2017). Scaling accurate genetic variant discovery to tens of thousands of samples. *BioRxiv*, 201178.](https://www.zotero.org/google-docs/?ShzSJ8)

[Procter, J. B., Carstairs, G. M., Soares, B., Mourão, K., Ofoegbu, T. C., Barton, D., Lui, L., Menard, A., Sherstnev, N., Roldan-Martinez, D., Duce, S., Martin, D. M. A., & Barton, G. J. (2021). Alignment of Biological Sequences with Jalview. Methods in Molecular Biology (Clifton, N.J.), *2231*, 203–224. https://doi.org/10.1007/978-1-0716-1036-7_13](https://www.zotero.org/google-docs/?ShzSJ8)

[Purcell, S., Neale, B., Todd-Brown, K., Thomas, L., Ferreira, M. A. R., Bender, D., Maller, J., Sklar, P., de Bakker, P. I. W., Daly, M. J., & Sham, P. C. (2007). PLINK: A Tool Set for Whole-Genome Association and Population-Based Linkage Analyses. *The American Journal of Human Genetics*, *81*(3), 559–575. https://doi.org/10.1086/519795](https://www.zotero.org/google-docs/?ShzSJ8)

[R Core Team, R. (2013). R: A language and environment for statistical computing.](https://www.zotero.org/google-docs/?ShzSJ8)

[Robinson, J. T., Thorvaldsdóttir, H., Winckler, W., Guttman, M., Lander, E. S., Getz, G., & Mesirov, J. P. (2011). Integrative genomics viewer. *Nature Biotechnology*, *29*(1), Article 1. https://doi.org/10.1038/nbt.1754](https://www.zotero.org/google-docs/?ShzSJ8)

[Salamov, A. A., & Solovyev, V. V. (2000). Ab initio gene finding in Drosophila genomic DNA. *Genome Research*, *10*(4), 516–522.](https://www.zotero.org/google-docs/?ShzSJ8)

[Sedlazeck, F. J., Rescheneder, P., Smolka, M., Fang, H., Nattestad, M., von Haeseler, A., & Schatz, M. C. (2018). Accurate detection of complex structural variations using single-molecule sequencing. *Nature Methods*, *15*(6), Article 6. https://doi.org/10.1038/s41592-018-0001-7](https://www.zotero.org/google-docs/?ShzSJ8)

[Seppey, M., Manni, M., & Zdobnov, E. M. (2019). BUSCO: Assessing genome assembly and annotation completeness. In *Gene prediction* (pp. 227–245). Springer.](https://www.zotero.org/google-docs/?ShzSJ8)

[Slater, G. S. C., & Birney, E. (2005). Automated generation of heuristics for biological sequence comparison. *BMC Bioinformatics*, *6*(1), 31. https://doi.org/10.1186/1471-2105-6-31](https://www.zotero.org/google-docs/?ShzSJ8)

[Smit, A. F. A., Hubley, R., & Green, P. (2015). RepeatMasker Open-4.0. 2015. Seattle, WA, USA.](https://www.zotero.org/google-docs/?ShzSJ8)

[Soltani, N., Best, T., Grace, D., Nelms, C., Shumaker, K., Romero-Severson, J., Moses, D., Schuster, S., Staton, M., Carlson, J., & Gwinn, K. (2020). Transcriptome profiles of *Quercus rubra* responding to increased O3 stress. *BMC Genomics*, *21*(1), 160. https://doi.org/10.1186/s12864-020-6549-5](https://www.zotero.org/google-docs/?ShzSJ8)

[Sork, V. L., Fitz-Gibbon, S. T., Zimin, A. V., Puiu, D., Garcia, J. A., Gugger, P. F., Henriquez, C. L., Zhen, Y., Lohmueller, K. E., Pellegrini, M., & Salzberg, S. L. (2022). High-quality genome and methylomes illustrate features underlying evolutionary success of oaks. *Nature Communications*, *13*(1), Article 1. https://doi.org/10.1038/s41467-022-29584-y](https://www.zotero.org/google-docs/?ShzSJ8)

[Tamura, K., Stecher, G., & Kumar, S. (2021). MEGA11: Molecular Evolutionary Genetics Analysis Version 11. *Molecular Biology and Evolution*, *38*(7), 3022–3027. https://doi.org/10.1093/molbev/msab120](https://www.zotero.org/google-docs/?ShzSJ8)

[Vaser, R., Sović, I., Nagarajan, N., & Šikić, M. (2017). Fast and accurate de novo genome assembly from long uncorrected reads. *Genome Research*, *27*(5), 737–746.](https://www.zotero.org/google-docs/?ShzSJ8)

[Verde, I., Abbott, A. G., Scalabrin, S., Jung, S., Shu, S., Marroni, F., Zhebentyayeva, T., Dettori, M. T., Grimwood, J., Cattonaro, F., Zuccolo, A., Rossini, L., Jenkins, J., Vendramin, E., Meisel, L. A., Decroocq, V., Sosinski, B., Prochnik, S., Mitros, T., … Rokhsar, D. S. (2013). The high-quality draft genome of peach (*Prunus persica*) identifies unique patterns of genetic diversity, domestication and genome evolution. *Nature Genetics*, *45*(5), 487–494. https://doi.org/10.1038/ng.2586](https://www.zotero.org/google-docs/?ShzSJ8)

[Waite, J. M., & Dardick, C. (2021). The roles of the IGT gene family in plant architecture: Past, present, and future. *Current Opinion in Plant Biology*, *59*, 101983. https://doi.org/10.1016/j.pbi.2020.101983](https://www.zotero.org/google-docs/?ShzSJ8)

[Wang, J., Tian, S., Sun, X., Cheng, X., Duan, N., Tao, J., & Shen, G. (2020). Construction of Pseudomolecules for the Chinese Chestnut (*Castanea mollissima*) Genome. *G3 Genes|Genomes|Genetics*, *10*(10), 3565–3574. https://doi.org/10.1534/g3.120.401532](https://www.zotero.org/google-docs/?ShzSJ8)

[Wu, T. D., & Nacu, S. (2010). Fast and SNP-tolerant detection of complex variants and splicing in short reads. *Bioinformatics*, *26*(7), 873–881. https://doi.org/10.1093/bioinformatics/btq057](https://www.zotero.org/google-docs/?ShzSJ8)

[Zomer, R. J., Trabucco, A., Bossio, D. A., & Verchot, L. V. (2008). Climate change mitigation: A spatial analysis of global land suitability for clean development mechanism afforestation and reforestation. *Agriculture, Ecosystems & Environment*, *126*(1), 67–80. https://doi.org/10.1016/j.agee.2008.01.014](https://www.zotero.org/google-docs/?ShzSJ8)
